# Efficient blood cell classification from microscopic smear images using U-Net segmentation and a lightweight CNN

**DOI:** 10.1038/s41598-025-26947-5

**Published:** 2025-12-27

**Authors:** Sohag Kumar Mondal, Md. Simul Hasan Talukder, Mohammad Aljaidi, Rejwan Bin Sulaiman, Md Mohiuddin Sarker Tushar, Amjad A. Alsuwaylimi

**Affiliations:** 1 Electrical and Electronic Engineering, Khulna University of Engineering and Technology, Khulna, Bangladesh; 2 Electrical and Electronic Engineering, Dhaka University of Engineering & Technology, Gazipur, Bangladesh; 3 Department of Computer Science, Faculty of Information Technology, Zarqa University, Zarqa, Jordan; 4 School of Computer science and Technology, Northumbria University, Newcastle-upon-Tyne, UK; 5 Electrical and Electronic Engineering, Bangabandhu Sheikh Mujibur Rahman Science and Technology University, Gopalganj, Bangladesh; 6 Department of Computer Science, College of Science, Northern Border University, Arar, Saudi Arabia

**Keywords:** Blood cell classification, BloodCell-Net, U-Net, Segmentation, Light weight CNN, Watershed algorithm, Computer science, Computational science, Biomedical engineering, Applied mathematics

## Abstract

Blood cell classification and counting are vital for the diagnosis of various blood-related diseases, such as anemia, leukemia, lymphoma, and thrombocytopenia. The manual process of blood cell classification and counting is time-consuming, prone to errors, and labor-intensive. Therefore, we have proposed a deep learning (DL)-based automated system for blood cell classification and counting from microscopic blood smear images. We classify a total of nine types of blood cells, including Erythrocyte, Erythroblast, Neutrophil, Basophil, Eosinophil, Lymphocyte, Monocyte, Immature Granulocytes, and Platelet. Several preprocessing steps like image resizing, rescaling, contrast enhancement and augmentation are utilized. To segment the blood cells from the entire microscopic images, we employed the U-Net model. This segmentation technique aids in extracting the region of interest (ROI) by removing complex and noisy background elements. Both pixel-level metrics such as accuracy, precision, and sensitivity, and object-level evaluation metrics like Intersection over Union (IOU) and Dice coefficient are considered to comprehensively evaluate the performance of the U-Net model. The segmentation model achieved impressive performance metrics, including 98.23% accuracy, 98.40% precision, 98.26% sensitivity, 95.97% Intersection over Union (IOU), and 97.92% Dice coefficient. Subsequently, a watershed algorithm is applied to the segmented images to separate overlapped blood cells and extract individual cells. We have proposed a BloodCell-Net approach incorporated with custom light weight convolutional neural network (LWCNN) for classifying individual blood cells into nine types. Comprehensive evaluation of the classifier’s performance is conducted using metrics including accuracy, precision, recall, and F1 score. The classifier achieved an average accuracy of 97.10%, precision of 97.19%, recall of 97.01%, and F1 score of 97.10%. A 5-fold cross-validation technique is applied to split the data, which not only aids in reducing overfitting but also helps in generalizing the model.

## Introduction

**Fig. 1:**
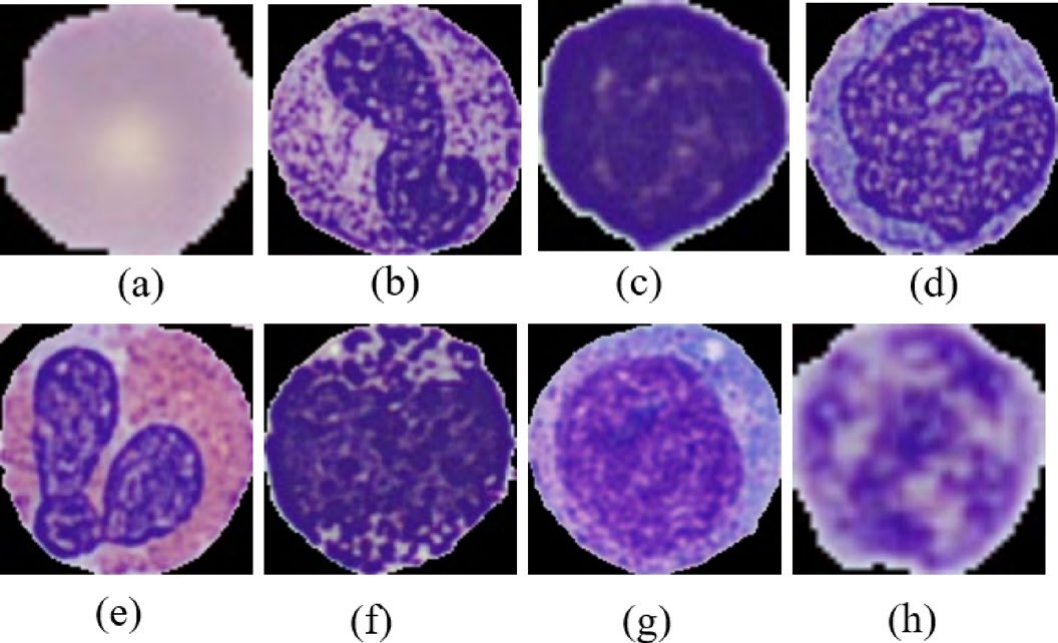
Different types of blood cells, (**a**) Erythrocyte (Red Blood Cell), (**b**) Neutrophils, (**c**) lymphocytes, (**d**)monocytes, (**e**) eosinophils, (**f**) basophils (**g**) Immature Granulocytes and (h) Thrombocytes (Platelets).

 Blood performs several vital functions in the body, including immune defense which serves as a defense mechanism against foreign elements and the transportation of oxygen, nutrients, and hormones. The blood contains cells and a portion known as plasma^1^. The blood cells comprise 45% of the total volume, while the liquid plasma constitutes the remaining 55%^2,3^. The blood cells can be classified as 3 class, red blood cells (RBC) or erythrocytes, white blood cell (WBC) or leukocytes and platelets or thrombocytes^4^. The RBCs account for 40–45% of the blood, whereas the WBCs constitute approximately 1% of the blood^5,6^. The various types of blood cells serve distinct roles within the body’s organs. RBCs are primarily responsible for transporting oxygen from the lungs to tissues and organs throughout the body, facilitated by the protein hemoglobin. Meanwhile, WBCs play a critical role in the immune system, defending against infections and foreign invaders. Neutrophils, lymphocytes (including T cells and B cells), monocytes, eosinophils, and basophils comprise the diverse array of WBCs, each specializing in various aspects of immune defense and regulation^6,7^. Basophils release histamine and heparin, which regulate allergic reactions and inflammation^8^. Eosinophils fight parasites and help regulate allergic responses. Monocytes mature into macrophages and dendritic cells, aiding in immune surveillance and presenting antigens. Lymphocytes, comprising T cells, B cells, and natural killer cells, play crucial roles in adaptive immunity^8^. Neutrophils quickly respond to bacterial infections, using phagocytosis and antimicrobial substances. A comprehensive understanding of these white blood cell types is crucial for diagnosing and treating immune-related conditions. It is observed that the proportions of neutrophils, eosinophils, lymphocytes, monocytes, and basophils in the blood are approximately 40–60%, 1–4%, 20–40%, 2–8%, and 0.5–1%, respectively^5^. Immature granulocytes, also known as band cells, are a type of WBC precursor that is produced in the bone marrow^9^. They represent an intermediate stage in the maturation of granulocytes, which include neutrophils, eosinophils, and basophils. Immature granulocytes are released into the bloodstream in response to infection or inflammation and are sometimes referred to as a “left shift” when their percentage in the blood is elevated. Platelets, also known as thrombocytes, are essential for blood clotting, which is crucial for wound healing and preventing excessive bleeding. Figure 1 illustrates the various types of blood cells. Together, these blood cells collaborate to maintain homeostasis, protect against pathogens, and ensure the proper functioning of bodily systems.

The peripheral blood smear is a standard laboratory examination that offers physicians extensive insights into a patient’s overall health status^10^. Leukemia and malaria are diseases characterized by alterations in WBC count, emphasizing the significance of early diagnosis^11^. Additionally, Patients can be evaluated for various health conditions, including immune system disorders and the presence of cancerous cells^12^. It offers both qualitative and quantitative evaluations of blood constituents, primarily focusing on cells and platelets. The manual blood cell count relies on the microscopic examination of the blood smear by the analyst, who distinguishes between subtypes primarily based on the morphological features including size, shape, texture, nucleus of the cell nucleus and cytoplasm^13^. Nonetheless, this process can be time-consuming and prone to errors if the microscopists are not adequately trained^14^. Furthermore, as this hematological assessment is a routine test, it frequently experiences high demand in clinical laboratories, leading to increased workload and impacting performance^10^. Thus, the implementation of a computer-aided diagnosis (CAD) system is necessary to provide diagnostic assistance in the laboratory.

The CAD system for blood cell classification can be decomposed as four parts, segmentation, ROI extraction, Feature Extraction and classification. Typically, cell segmentation poses a challenge in tissue samples. However, this process is simpler in cell smears due to the distinct appearance of the dark nucleus in staining blood smear. Performing segmentation before classification in blood cell classification tasks offers several advantages. It enhances accuracy through the isolation of individual cells and extraction of specific features, thereby improving classification and counting capabilities. This approach reduces computational complexity and enhances robustness by eliminating backgrounds and non-essential substances. Overall, segmentation before classification enhances the accuracy, efficiency, and robustness of blood cell classification tasks. In traditional image segmentation techniques, including manual thresholding^15^, OTSU binarization^16^, fuzzy c-means (FCM)^17^, active contours^18^, and watershed algorithm^19^, are employed. Following the advent of deep learning (DL), numerous segmentation techniques have been developed, including the fully connected network (FCN)^20^, U-Net^21^, Faster-RCNN^22^, YOLO^23^, among others. These approaches have demonstrated superior performance compared to traditional methods for blood cell segmentation.

In conventional image processing techniques, the images are classified based on different features like histogram of gradients (HOG), colors, texture, geometric, edges, and statistical features^24^. Due to the benefits of artificial intelligence in image analysis, various machine learning (ML) techniques have been examined for the classification and segmentation of leukocytes. These approaches span from conventional methods like support vector machines (SVM)^25^ and Naïve Bayesian^26^ to advanced algorithms such as DL models^27,28^. Transfer learning has introduced an additional dimension to the classification of blood cells by accelerating the training speed. Researchers utilized different transfer learning models like AlexNet^29^, ResNet^30^, VGG^27^, GoogLeNet^31^ for automated blood cell classification.

This study presents a novel BloodCell-Net scheme for the segmentation and classification of nine types of blood cells, including Erythrocyte, Erythroblast, Neutrophil, Basophil, Eosinophil, Lymphocyte, Monocyte, Immature Granulocytes, and Platelet. We utilized various data preprocessing techniques, including image resizing, normalization, histogram equalization and augmentation. These preprocessing steps enhance model performance during data preparation. Image resizing ensures uniformity in image dimensions, aiding efficient processing. Normalization of pixel values stabilizes training and improves the model’s learning ability. Histogram equalization enhances feature visibility for better extraction. Augmentation techniques, such as rotation and scaling, increase dataset diversity, reducing overfitting. Together, these steps optimize the dataset for robust model training. To segment the blood cells from the background, we employed the U-Net model, specifically designed for segmenting medical images. The pixel-wise segmentation model enhances classifier performance by isolating the non-important background from the region of interest (ROI). In microscopic thin blood smear images, the overlapping of blood cells is a common occurrence, presenting challenges in their separation. Hence, the Watershed algorithm is utilized to effectively separate overlapping cells and extract the ROIs from the segmented images. Here in our study the ROIs are the single blood cells. Finally, we proposed a custom sequential LWCNN architecture to classify the blood cells. The contribution of this research work is given below.

- Preparation of pre-processed dataset having nine types of blood smear dataset.
- Introducing data scaling, histogram equalization & augmentation.
- Implementing a precise segmentation approach to accurately delineate the regions of interest within the images.
- Application of the watershed algorithm to separate overlapping cells and extract the ROIs from segmented images.
- Proposing an effective LWCNN architecture for performing classification tasks.
- Presenting a novel BoodCell-Net approach to detecting nine types of blood cells.
- Evaluation of the model with well-known performance metrics.

The proposed paper is structured as follows: Sect. 1 presents the introduction, while Sect. 2 provides a review of the literature. The proposed framework model is detailed in Sect. 3, with the results and discussion presented in Sect. 4. Finally, Sect. 5 offers the conclusion.

## Literature review

Artificial intelligence is an emerging technology that is being used widely in agriculture^32^, medical image processing^33^, and healthcare^34^ for diagnostic purposes as well as data quality enhancement by removing noise using several techniques such as contrastive semi-supervised networks (CS-Net)^35^. Classification techniques such as traditional machine learning, random forests, decision trees, and so on, tuned regularized absolute network-based logistic regression models^36^, extreme machine learning^37^, ensemble learning^38^, convolutional neural networks, and transfer learning^39^ are widely used. Precisely following the research, the community extensively employs both traditional machine learning (TML) and deep learning (DL) models for the automated classification of blood cells^40,10,41,42^. Many researchers divide this problem into two parts, segmentation and classification, in order to find the appropriate solution^43,44^. Segmentation enhances classifier performance by removing non-relevant parts from the images. Segmentation techniques, such as thresholding, morphological operations, and machine learning, Unet, pre-trained Unet, DL-based ULM, AM-Net^45^ are commonly employed in research cited as^46–51^. Nee et al. introduced a segmentation technique for WBCs in acute leukemia bone marrow images, leveraging thresholding and morphological operations^50^. The research article cited as^49^ implemented a segmentation process for blood cells utilizing thresholding and Canny edge detection, followed by enhancing local and global details of the output through morphological operations. The authors claimed an average segmentation accuracy of 87.9% in the public human RBC dataset. Both segmentation and classification of WBCs are performed by Pešić to detect Acute Lymphoblastic Leukemia. The author employed the Otsu thresholding technique twice on the H channel in the HSI color space. In machine learning for blood cell segmentation both supervised and unsupervised techniques are used^46,47,52,53^. Tran et al. introduced a CNN based model called SegNet for the classification of blood cells. Notably, this model was initialized with weights derived from the VGG-16 network. Among the three types of blood cells, the researchers considered RBCs and WBCs, while omitting platelets from their analysis. U-Net, a specialized CNN-based model designed specifically for medical image segmentation, was utilized by Zhang et al. in their article^52^. The proposed deformable U-Net (dU-Net) demonstrated superior performance on both binary segmentation and multiclass semantic segmentation tasks^52^. Besides supervised ML models, the unsupervised ML models are also used among researcher communities for the blood cell segmentation^47,53^. The research cited as^47,53^ is utilized and discusses k-means clustering image segmentation technique to segment the WBCs in their paper. Zheng et al. in^53^, proposed a novel technique for separating the overlapping cells after segmentation based on the touching-cell clump splitting technique.

In addition to microscopy-specific pipelines, several adjacent advances can strengthen segmentation-first CAD systems. Multimodal self-supervised pretraining—e.g., a Multimodal Masked Autoencoder with adaptive masking and a reconstruction pretext task—reduces dependence on scarce annotations and improves downstream classification when labeled multimodal data are limited, suggesting a path to more label-efficient hematology models^54^. Modern deep-learning denoisers (e.g., CS-Net for ultrasound localization microscopy) report sizable SNR/CNR gains while achieving practical per-frame runtimes, illustrating that learned denoising can both enhance image quality and meet lab-throughput constraints relevant to smear analysis^35^. On the acquisition side, recent objective-lens designs target sub-micron resolution alongside a much larger field of view than conventional sub-micron objectives, indicating how higher-context, higher-quality inputs can reduce overlap and stitching artifacts that complicate instance separation^55^. More broadly, advances in precision instrumentation and biosensing (e.g., optically pumped magnetometry), rigorous clinical study design and translational reporting standards^56,57^, and single-cell systems biology that maps cell-state heterogeneity^58^ provide useful context for dataset curation, validation endpoints, and error analysis when developing CAD pipelines for hematology. Complementing these trends, neuromorphic, video-activated imaging-flow cytometry shows that pairing event-camera acquisition with spiking neural networks can sustain ~ 1000 cells/s sorting and substantially improve RBC/spherocyte discrimination versus single-frame decisions, highlighting the promise of spatiotemporal cues for robust hematology CAD^59^.

Classification plays a pivotal role in comprehending the blood profile and estimating cell density by counting the number of cells. Some authors have employed traditional machine learning techniques, while others have utilized deep learning approaches such as custom CNNs, transfer learning, and hybrid models for blood cell classification^60,10,61,11^. Hegde et al. introduced a leukemia detection system employing an SVM classifier^25^. The authors incorporated various features such as shape, color, and texture into their analysis. The proposed approach yields encouraging outcomes in differentiating between normal and abnormal WBCs, potentially reducing the workload for pathologists significantly. An automatic leukocytes classification system is built by extracting morphological features based on the Naïve Bayes Classifier by Gautam et al.^62^. The features which are extracted by the authors are area, eccentricity, perimeter and circularity of leukocyte nucleus. This research article claimed an accuracy of 80.88% for this particular task. On the contrary, Prinyakupt et al. employed and evaluated the performance of two distinct models, namely the linear classifier and Naïve Bayes, for the WBC classification system^26^. They show that the linear classifier outperforms the Naïve Bayes.

In addition to traditional machine learning techniques, researchers have also incorporated deep learning models into the task of blood cell classification^60,63–66^. A new 33-layer CNN model called WBCNet model that can extract features and classify WBCs from microscopic WBC images is proposed by Jiang et al.^60^. This research integrated the batch normalization algorithm, residual convolution architecture, and enhanced activation functions to enhance the classification score. The study referenced as^64^ proposes a CNN-based approach utilizing Genetic Algorithm (GA) to classify four types of leukocytes, achieving 99% training accuracy and 91% validation accuracy. Su et al. highlighted and extracted three types of features called geometrical features, color features, and LDP-based texture features for WBC classification^67^. The extracted features are feeded into three different kinds of neural networks to recognize the types of the WBCs. The simulation results demonstrated that the proposed system for classifying WBCs is highly competitive when compared to several existing systems. A Regional Based CNN (R-CNN) is proposed by Kutlu et al. for classifying WBCs^65^. The proposed model also compared with four transfer learning models called AlexNet, VGG16, GoogLeNet, ResNet50, and claimed the proposed R-CNN outperformed the state-of-the-arts. Feature fusion is the process of combining information from multiple sources or representations into a single representation to improve performance^68^. A novel feature fusion based deep learning framework for white blood cell classification is proposed by Dong et al.^69^. The authors fused deep learning features with artificial features to generate the final features for WBC classification.

Various transfer learning models such as AlexNet, MobileNetV2, DenseNet161, and DarkNet-53 are employed for the classification of blood cells and leukemia^70–74^. Loey et al. utilized a pre-trained deep CNN, namely AlexNet, to extract features before classifying WBCs as either healthy or infected by leukemia^70^. Tamang et al. assessed various state-of-the-art models using multiple evaluation metrics such as accuracy, precision, recall, F1 score and training time ultimately recommending DenseNet161 for blood cell classification^71^. Furthermore, advanced optimization methods like normalization, mixed-up augmentation, and label smoothing were applied to DenseNet to further enhance its performance. In another study, Yang et al. trained three distinct CNN architectures by initializing the pretrained parameters on ImageNet and recommended MobileNetV2 for blood cell classification^74^. This research claimed 89.40% accuracy and 91.60% precision on the BCCD dataset. The study conducted by Saleem et al. employed a generative adversarial network (GAN) to augment image data for subsequent classification using DarkNet-53^73^. Less important features are dropped, and the more important features are selected using principal component analysis (PCA) technique. Subsequently, these features are fused using various machine learning models.

Oumaima Saidani et al.^75^ introduced one optimized CNN and four transfer learning models, namely MobileNetV2, VGG16, InceptionV3, and ResNet50 to classify five types of human blood cells collected from the IEEE data port. The study also integrated color conversion and augmentation preprocessing. The optimized CNN model achieved 99.86% accuracy.

Recently, Karnika Dwivedi et al.^76^ proposed a CNN-based architecture called MicrocellNet to classify eight types of blood cells. Their approach achieved 98.76% validation accuracy and 97.65% test accuracy. Similarly, another most recent work was carried out on eight types of blood cell classification by the researcher named Hüseyin Fırat et al.^77^. The author presented a hybrid method combination of the Inception module, pyramid pooling module (PPM), and depth-wise squeeze-and-excitation block (DSEB) to classify blood cells. The study used three datasets named four classes (BCCD dataset), five classes (Raabin WBC dataset), and eight classes and achieved 99.96%, 99.22%, and 99.72% accuracy, respectively. However, they did not use K-fold validation to check the method’s robustness.

Table 1 provides a detailed finding of the study survey across several literature reviews, highlighting similarities, differences, and potential gaps in the existing research. In the literature survey, three types of public datasets have been found that are used in different studies to classify blood cells in the human body. The datasets are four classes (BCCD), which have eosinophils, lymphocytes, monocytes and neutrophils; five classes (WBC) having Neutrophils, Basophils, Eosinophils, Monocytes and Lymphocytes; and eight classes Mendeley dataset having Erythroblast, Neutrophil, Basophil, Eosinophil, Lymphocyte, Monocyte, Immature Granulocytes, and Platelet. Notably, erythrocyte identification is excluded in all the research, but the number of erythrocytes in the blood must be counted, which is crucial for carrying oxygen throughout the body. Therefore, this test helps diagnose conditions like anemia (low red blood cell count) or other issues related to blood cell production or destruction, like internal bleeding, kidney disease, or bone marrow disorders. This comprehensive study has filled the scientific void of CAD-based identifying erythrocytes and other blood cells. Most studies concentrated on deep learning and transfer learning to classify blood cells. In contrast, a small study utilized statistical features and was classified by the traditional machine learning approach. The traditional statistical-based approach failed to achieve the desired performances. The deep learning approach requires huge trainable parameters and has issues of performance as well. Many of the studies have also spiked the robustness analysis for identifying blood cells. Fine-tuning of different preprocessing techniques, such as image quality enhancement using histogram equalization, segmentation for extracting the core part of the features, augmentation for diversifying datasets, and lightweight CNN, have also not been employed in any previous work.

**Table 1:** Comparative literature review summary.

Ref.	Classifier	Dataset	Accuracy
^78^	AlexNet, VGG Net 13, 11, ResNet 18, 34, 50, SqueezeNet 10, 11, DenseNet 121, 161.	BCCD dataset (four classes)	100% (DenseNet 161)
^79^	DT, RF, SVM, k-NN, CNN, VGG16, ResNet15	IEEE data port (3539images) Five classes	97% (RF)
^80^	CNN, ELM, AlexNet, GoogleNet, VGG-16, and ResNet	BCCD dataset (four classes)	96.03%
^81^	CNN	Kaggle	98.55%
^75^	Optimized CNN model	IEEE data port (3539images) (Five classes)	99.86%
^76^	CNN based MicrocellNet	Mendeley dataset (Eight classes)	97.65%
^77^	Hybrid method combination of the Inception module, pyramid pooling module (PPM), and depth wise squeeze-and-excitation block	1. Mendeley dataset (Eight classes) 2. IEEE data port (3539images) (Five classes) 3. BCCD dataset (four classes)	1. 99.72% 2. 99.22%, 3. 99.96%

In our study, we addressed all the points by preparing nine types of datasets, adding erythrocytes incorporation, advanced preprocessing techniques, and a lightweight CNN with suppressive performance.

## Materials and methods

**Fig. 2:**
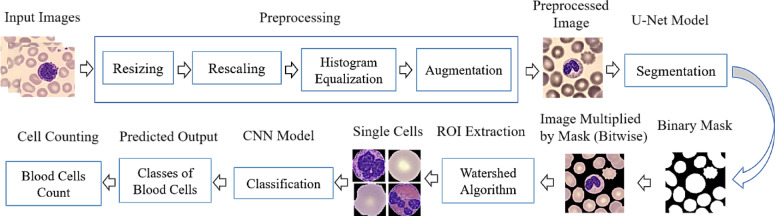
Block diagram of the proposed BloodCell-Net framework for blood cell classification.

In this section, we introduce the proposed BloodCell-Net framework for the classification of blood cells into nine distinct classes, as depicted in Fig. 2. The framework comprises several fundamental components, namely data preprocessing, segmentation, ROI extraction, and classification. Each of these components is elaborated upon sequentially as follows.

### Dataset description

The microscopic peripheral blood cell images dataset has been collected from a trustworthy Mendeley repository^82^. The dataset consists of 17,092 images of distinct cells of normal function which were obtained in the Hospital Clinic of Barcelona’s Core Laboratory using the CellaVision DM96 analyzer. It is categorized into eight groups: neutrophils, eosinophils, basophils, lymphocytes, monocytes, immature granulocytes (promyelocytes, myelocytes, and metamyelocytes), erythroblasts and platelets or thrombocytes. Expert clinical pathologists annotated the 360 × 363-pixel JPG photos. The blood samples were taken from people who were clear of infections, hematologic or oncologic diseases, and pharmaceutical treatments at the time of the photo session.

**Fig. 3:**
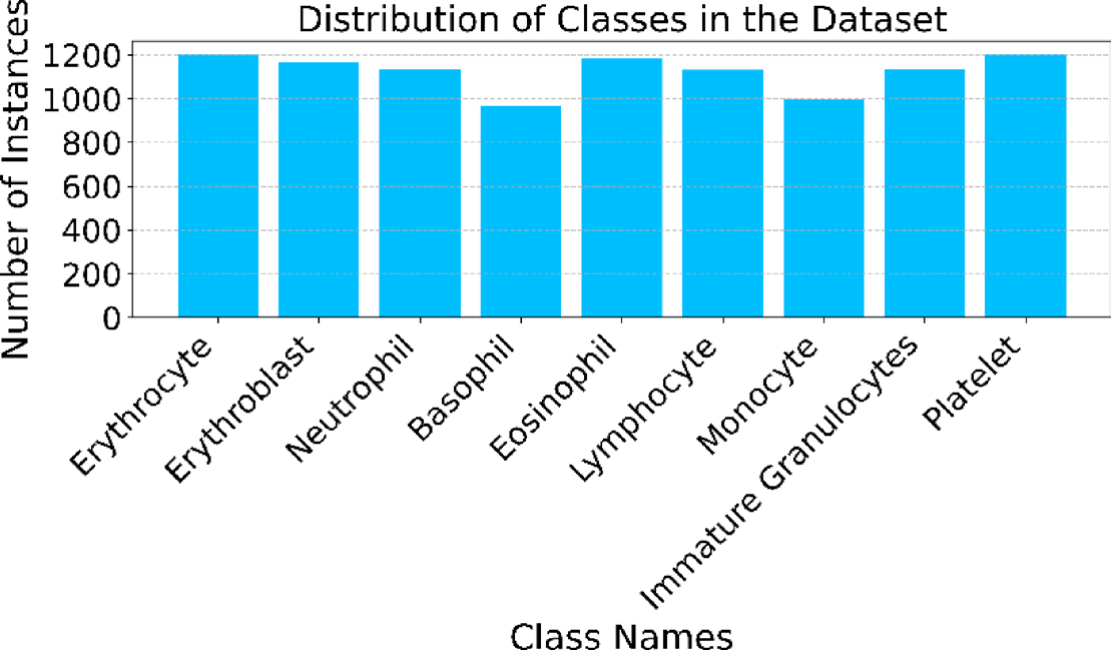
Bar plot for dataset distribution.

In this study, we extracted erythrocyte cells from the whole dataset using a watershed approach, which we named after the erythrocyte class. So, the dataset of our study consists of nine types of microscopic peripheral blood cells namely, Erythrocyte, Erythroblast, Neutrophil, Basophil, Eosinophil, Lymphocyte, Monocyte, Immature Granulocytes, and Platelet. The distribution and visual appearance of the dataset is shown in Table 2; Fig. 3 respectively.

**Table 2:** Distribution of images in each class.

Name of the class	Number of the images
Erythrocyte	1200
Erythroblast	1168
Neutrophil	1133
Basophil	969
Eosinophil	1186
Lymphocyte	1131
Monocyte	999
Immature Granulocytes	1134
Platelet	1204

### Preprocessing

Image preprocessing enhances model performance by improving input quality. We apply resizing for uniform dimensions, rescaling for normalization, histogram equalization for contrast enhancement, and augmentation (e.g., rotation, flipping) to increase data diversity. These steps enhance feature extraction, reduce overfitting, and improve classification accuracy.

#### Image resizing

In the dataset, we encountered images with varying sizes, which are not conducive to further processing. Therefore, we resize the images to 224 × 224 px to facilitate mini-batch learning, preserve computational restrictions, and be compatible with the available pre-trained models. Linear interpolation techniques have been employed in this study. The mathematical expression of image resizing by linear interpolation is given by the following equation-

1 $$I_{{resized}} \left( {x^{\prime } ,y^{\prime } } \right) = \left( {1 - a} \right).I_{{orginal}} \left( {x_{1} ,y^{\prime } } \right) + a.I_{{orginal}} \left( {x_{2} ,y^{{^{\prime } }} } \right)$$

Where, ɑ= fractional part of $\:{x}^{{\prime\:}}$ and $\:{x}_{1},{y}_{1}$ are the neighboring pixel indices. $\:{I}_{resized}\left({x}^{{\prime\:}},{y}^{{\prime\:}}\right)$ is the pixel intensity of the resized image and $\:{I}_{orginal}\left(x,{y}^{{\prime\:}}\right)$ is the pixel intensity of original image.

#### Rescaling

Rescaling or normalization in image data preprocessing is crucial for ensuring that pixel values across images are on a similar scale. This process is essential for machine learning models, particularly deep neural networks, as it prevents certain features or channels from dominating others during training. Normalization also helps in stabilizing and accelerating the convergence of optimization algorithms, such as gradient descent, by ensuring that gradients are not disproportionately influenced by large pixel values. Furthermore, normalization aids in improving the interpretability of CNN architecture by facilitating more consistent feature representations across layers, thus enabling better generalization and performance on image classification, segmentation, and other computer vision tasks. As the images in our dataset are RGB images, the pixel values range from 0 to 255. Therefore, we divided the pixel values by 255 to normalize them and bring all pixels within the range of 0 to 1. The expression normalization can be expressed by the following equation-

2 $$\:{x}^{{\prime\:}}=\frac{x-{x}_{min}}{{x}_{max}-x}$$

*x* is the original value and $\:{x}^{{\prime\:}}\:$is normalized value.

#### Histogram equalization

The images in the dataset are captured under various light conditions, leading to variations in brightness and contrast. That is why an image enhancement technique is required to equalize the histogram of the images. We utilized Contrast Limited Adaptive Histogram Equalization (CLAHE) technique to enhance image contrast by dividing the image into smaller regions, known as tiles, and applying histogram equalization to each tile independently^83^. This adaptive approach ensures that contrast enhancement is tailored to the local characteristics of the image, making it effective for images with non-uniform illumination or regions of varying contrast. To prevent over-amplification of noise in low-contrast areas, CLAHE incorporates a contrast limiting mechanism that clips the histogram beyond a specified threshold. Additionally, bilinear interpolation is used to blend adjacent tiles, smoothing out any discontinuities and artifacts.

#### Augmentation

**Fig. 4:**
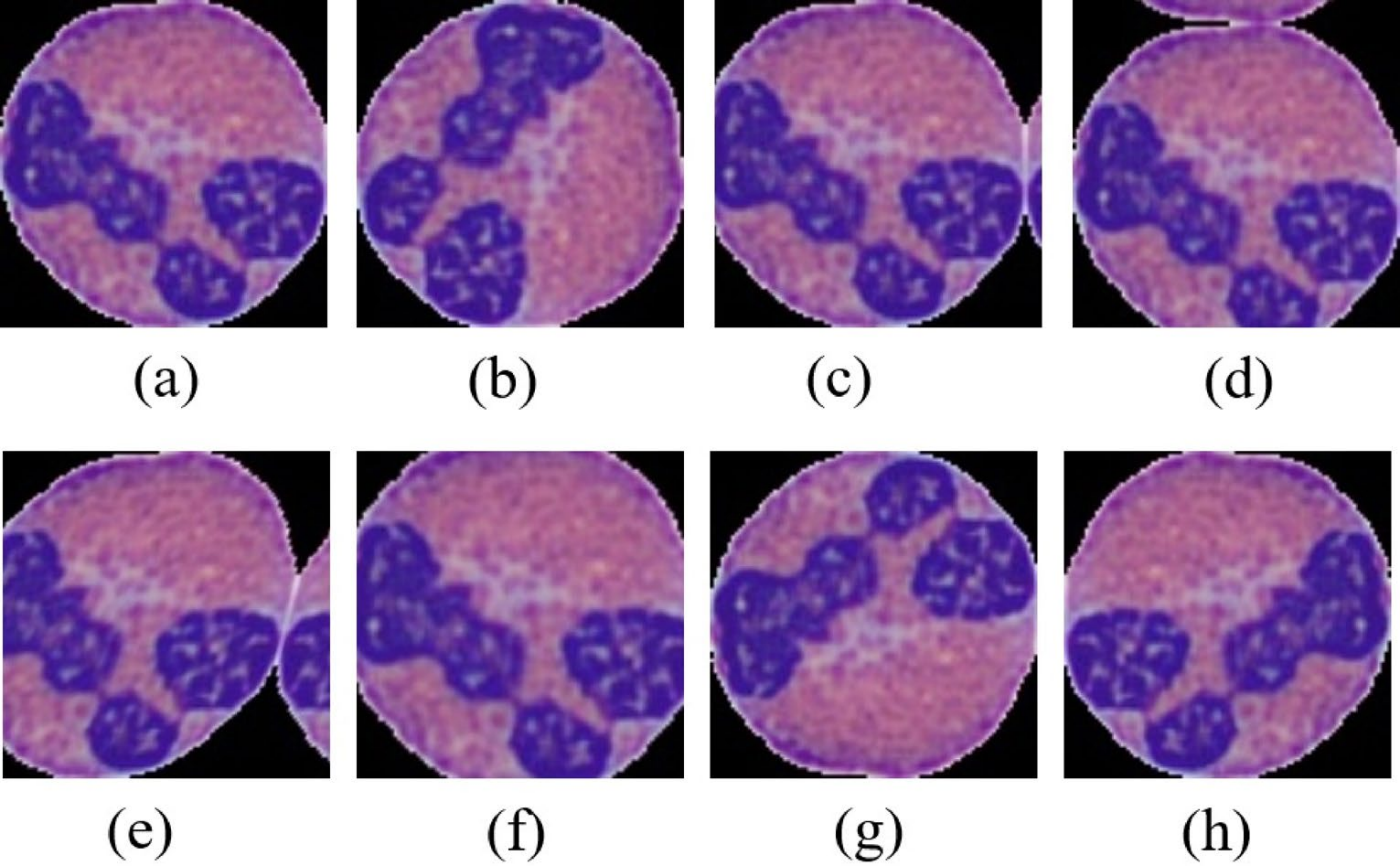
The visualization of image augmentation (**a**) original image, (**b**) rotation, (**c**) width shift, (**d**) height shift, (**e**) shear, (**f**) zoom, (**g**) horizontal flip and (**h**) vertical flip.

Augmentation is a technique to increase the number of training data from the existing ones using different morphological operations. In this study, seven augmentation strategies were applied that are rotation, width shift, height shift, shear, zoom, flip, and fill. The visualization of the blood cell after augmentation is depicted in Fig. 4.

##### Rotation

To reduce the model’s sensitivity to object positioning, random adjustments were made to the image angles, which ranged from − 20 to 20 degrees. This tactic facilitates the model’s ability to identify features across a range of orientations. A 2D affine transformation can be used to depict the rotation of an image.

3 $$\left[ {\begin{array}{*{20}c} {x^{\prime}} \\ {y^{\prime}} \\ \end{array} } \right] = \left[ {\begin{array}{*{20}c} {\cos \left( \theta \right)} & { - \sin \left( \theta \right)} \\ {\sin ~\left( \theta \right)} & {\cos \left( \theta \right)} \\ \end{array} } \right]\left[ {\begin{array}{*{20}c} x \\ y \\ \end{array} } \right]$$

Where, $\:\theta\:$ is the angle of rotation $\:(x,y$) are the initial coordinates in the image and $\:{(x}^{{\prime\:}},{y}^{{\prime\:}})\:$are the coordinates following rotation.

##### Width and height shift

Both height shift and width shift entail translating the image both vertically and horizontally. These modifications assist the model in adjusting to shifts in the locations of items within the frame.

These transformations have the following mathematical expression:

4 $$\:\left[\begin{array}{c}{x}^{{\prime\:}}\\\:{y}^{{\prime\:}}\end{array}\right]=\left[\begin{array}{c}x\\\:y\end{array}\right]+\left[\begin{array}{c}\varDelta\:x\\\:\varDelta\:y\end{array}\right]$$

In this expression, $\:(x,y)$ are the main coordinates in the image, $\:{(x}^{{\prime\:}},{y}^{{\prime\:}})\:$are the coordinates after employing the width and height shift transformations, and $\:\varDelta\:x,\varDelta\:y$ are the shift values for width and height, respectively. Adjusts from − 0.05 to 0.05, adds randomness, normalizes based on the size of the input image, and facilitates the model’s ability to adjust to new data.

##### Shear

The model’s flexibility is increased in response to minute modifications that occur in real-world data by applying shear transformations between 0 and 0.05. Shearing is shown as follows:

5 $$\:\left[\begin{array}{c}{x}^{{\prime\:}}\\\:{y}^{{\prime\:}}\end{array}\right]=\left[\begin{array}{cc}1&\:\lambda\:\\\:0&\:1\end{array}\right]\left[\begin{array}{c}x\\\:y\end{array}\right]$$

In this equation, (x, y) are the initial coordinates in the image, $\:{(x}^{{\prime\:}},{y}^{{\prime\:}})\:$are the coordinates following the shear transformation, and $\:\lambda\:$ is the shearing factor, ranging from 0 to 0.05.

##### Zoom

To allow the model to adapt to variations in object size, zoom transformations between 0 and 0.05 have been added. This enhances the model’s perception of features at varying magnifications. The act of zooming is defined as:

6 $$\left[ {\begin{array}{*{20}c} {x^{\prime}} \\ {y^{\prime}} \\ \end{array} } \right] = \left[ {\begin{array}{*{20}c} \alpha & 0 \\ 0 & \beta \\ \end{array} } \right]\left[ {\begin{array}{*{20}c} x \\ y \\ \end{array} } \right]$$

where $\:(x,y)$ are the initial coordinates in the image, $\:{(x}^{{\prime\:}},{y}^{{\prime\:}})\:$are the coordinates following employing the zoom transformation, and $\:(\alpha\:,\beta\:)$ are the scaling factors for width and height, respectively, which vary from 1 to 1.2 to indicate a zoom range of 0 to 0.05.

##### Horizontal flip

To increase the model’s comprehension of symmetry, a horizontal flip augmentation is used. As a result, the model can identify features both in their original and reversed states. A straightforward process called “horizontal flipping” flips the values of the pixels along the vertical axis.

7 $$\:{I}_{flipped}\left(x.y\right)=I(-x,y)$$

The pixel value at location $\:(x,y)$ in the flipped image is represented by the equation $\:{I}_{flipped}(x,y)$ and the pixel value at the mirrored position in the original image is indicated by $\:I(-x,y).$.

##### Fill mode

“Fill Mode” is a technique for managing portions of a picture that could appear after geometric operations like rotation, shifting, or shearing. After an image has been converted, if some pixels are not filled with their original values, there can be blank or undefinable parts. For a pixel at coordinates $\:{(x}^{{\prime\:}},{y}^{{\prime\:}})\:$ in the transformed image, the corresponding pixel value $\:{I(x}^{{\prime\:}},{y}^{{\prime\:}})\:$can be approximated using nearest-neighbor interpolation as

8 $$\:{(I}^{{\prime\:}}\left({(x}^{{\prime\:}},{y}^{{\prime\:}}\right)=I\left(\right|{(x}^{{\prime\:}}|,|{y}^{{\prime\:}}\left|\right)$$

### Image segmentation

**Fig. 5:**
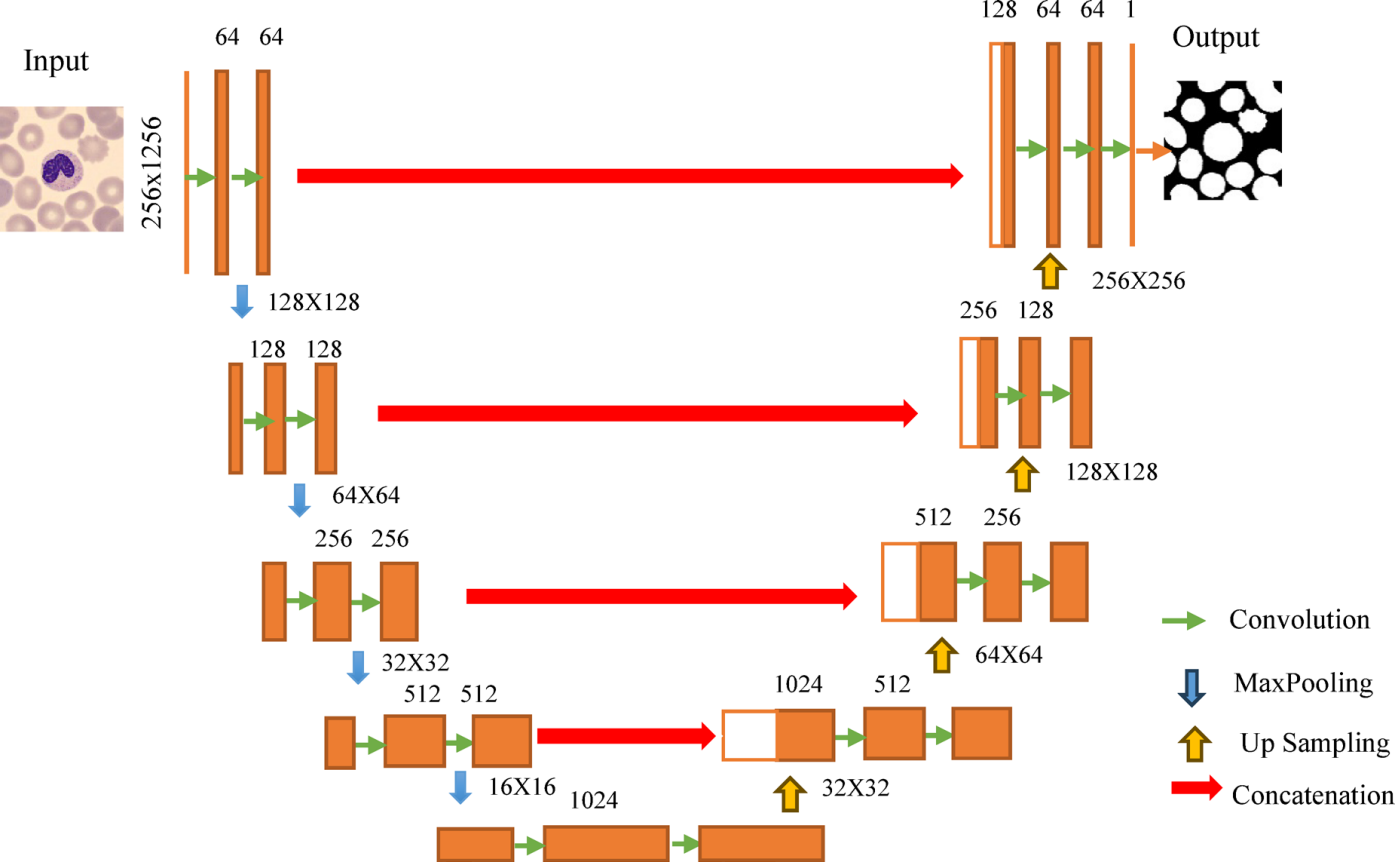
Architecture of U-Net model.

In this study, a U-Net model is deployed to segment the peripheral blood cell images. The U-Net architecture leverages encoder–decoder skip connections and multi-resolution feature fusion to achieve accurate segmentation of microscopic blood cell images. By preserving spatial details and capturing hierarchical features, the model demonstrates robust performance in segmenting complex cell structures, contributing to advancements in medical image analysis. The encoder portion of the network consists of four convolutional blocks followed by max-pooling layers to down-sample the spatial dimensions of the input image while increasing the number of feature maps. Each convolutional block applies two 3 × 3 convolutional layers with ReLU activation, preserving spatial information and extracting increasingly abstract features. The number of filters doubles with each block, starting from 64 and ending with 512. Following the encoder, a middle convolutional block further processes the feature maps, aiming to capture high-level semantic information. This block has the same structure as the previous ones but operates on the highest resolution feature maps extracted by the encoder. The decoder portion of the network mirrors the encoder’s structure, but with up-sampling operations to gradually restore the spatial dimensions of the feature maps. After each up-sampling operation, concatenation is performed between the up-sampled feature maps and the corresponding feature maps from the encoder, enabling the network to recover spatial details lost during down-sampling. Each concatenated feature map undergoes convolutional operations to refine the segmentation output. The number of filters decreases with each block, mirroring the pattern of the encoder. The final layer of the network consists of a 1 × 1 convolutional layer with sigmoid activation, producing the segmentation mask for the input image. The sigmoid activation function ensures that the output values are in the range [0, 1], representing the probability of each pixel belonging to the foreground class (blood cell). For training and tuning, inputs were resized to 256 × 256 with per-image normalization; weights were He-initialized and trained with Adam (learning rate 1 × 10⁻³, β₁=0.9, β₂=0.999, ε = 1 × 10⁻⁷), binary cross-entropy as the loss, and batch size 16. We employed early stopping (patience = 10 epochs, restoration of best weights) and ReduceLROnPlateau (factor = 0.5, patience = 5, LR floor = 1 × 10⁻⁵). Lightweight L2 kernel regularization (1 × 10⁻⁴) was applied to convolutional layers, and standard on-the-fly augmentation (random rotation/shift/flip/zoom within the ranges specified in Sect. 3.2) was used consistently across folds. A brief ablation during tuning indicated that adding a Dice term to the loss or replacing ReLU with GELU did not yield consistent gains on validation IoU/Dice; therefore, the above configuration was retained for the final model reported in Fig. 5; Table 3. The model is trained using the binary cross-entropy loss function and the Adam optimizer. Binary cross-entropy is suitable for binary classification tasks like image segmentation, where each pixel is classified as either foreground (blood cell) or background. In Fig. 5 the structure of U-Net architecture is depicted, where Table 3 is constructed with the architecture of U-Net model.

**Table 3:** Summary table of U-Net architecture.

Layer (Type)	Feature Map	No. of Parameters	Kernel Size	Activation Function
InputLayer	(None, 256, 256, 3)	0	-	-
Conv2D_1	(None, 256, 256, 64)	1792	(3, 3)	ReLU
Conv2D_2	(None, 256, 256, 64)	36,928	(3, 3)	ReLU
MaxPooling2D_1	(None, 128, 128, 64)	0	(2, 2)	-
Conv2D_3	(None, 128, 128, 128)	73,856	(3, 3)	ReLU
Conv2D_4	(None, 128, 128, 128)	147,584	(3, 3)	ReLU
MaxPooling2D_2	(None, 64, 64, 128)	0	(2, 2)	-
Conv2D_5	(None, 64, 64, 256)	295,168	(3, 3)	ReLU
Conv2D_6	(None, 64, 64, 256)	590,080	(3, 3)	ReLU
MaxPooling2D_3	(None, 32, 32, 256)	0	(2, 2)	-
Conv2D_7	(None, 32, 32, 512)	1,180,160	(3, 3)	ReLU
Conv2D_8	(None, 32, 32, 512)	2,359,808	(3, 3)	ReLU
MaxPooling2D_4	(None, 16, 16, 512)	0	(2, 2)	-
Conv2D_9	(None, 16, 16, 1024)	4,719,616	(3, 3)	ReLU
Conv2D_10	(None, 16, 16, 1024)	9,438,208	(3, 3)	ReLU
UpSampling2D_1	(None, 32, 32, 1024)	0	(2, 2)	-
Concatenate_1	(None, 32, 32, 1536)	0	-	-
Conv2D_11	(None, 32, 32, 512)	7,078,400	(3, 3)	ReLU
Conv2D_12	(None, 32, 32, 512)	2,359,808	(3, 3)	ReLU
UpSampling2D_2	(None, 64, 64, 512)	0	(2, 2)	-
Concatenate_2	(None, 64, 64, 768)	0	-	
Conv2D_13	(None, 64, 64, 256)	1,769,728	(3, 3)	ReLU
Conv2D_14	(None, 64, 64, 256)	590,080	(3, 3)	ReLU
UpSampling2D_3	(None, 128, 128, 256)	0	(2, 2)	-
Concatenate_3	(None, 128, 128, 384)	0	-	-
Conv2D_15	(None, 128, 128, 128)	442,496	(3, 3)	ReLU
Conv2D_16	(None, 128, 128, 128)	147,584	(3, 3)	ReLU
UpSampling2D_4	(None, 256, 256, 128)	0	(2, 2)	-
Concatenate_4	(None, 256, 256, 192)	0	-	-
Conv2D_17	(None, 256, 256, 64)	110,656	(3, 3)	ReLU
Conv2D_18	(None, 256, 256, 64)	36,928	(3, 3)	ReLU
Conv2D_19	(None, 256, 256, 1)	65	(1, 1)	Sigmoid

The U-Net model generates a binary mask, where the background is black (0) and the blood cells are white (1). By performing bitwise multiplication between this mask and the original image, the background pixels, having a value of 0, are completely removed, effectively eliminating complex and noisy elements from the microscopic images. To further refine segmentation, we apply the Watershed algorithm, which is well-suited for separating overlapping cells. By treating the binary mask as a topographic surface, Watershed ensures precise delineation of individual cells. This combination of U-Net’s deep learning-based segmentation and Watershed’s contour-based separation enhances accuracy in extracting ROIs, making our approach robust and effective.

### Watershed algorithm

**Fig. 6:**
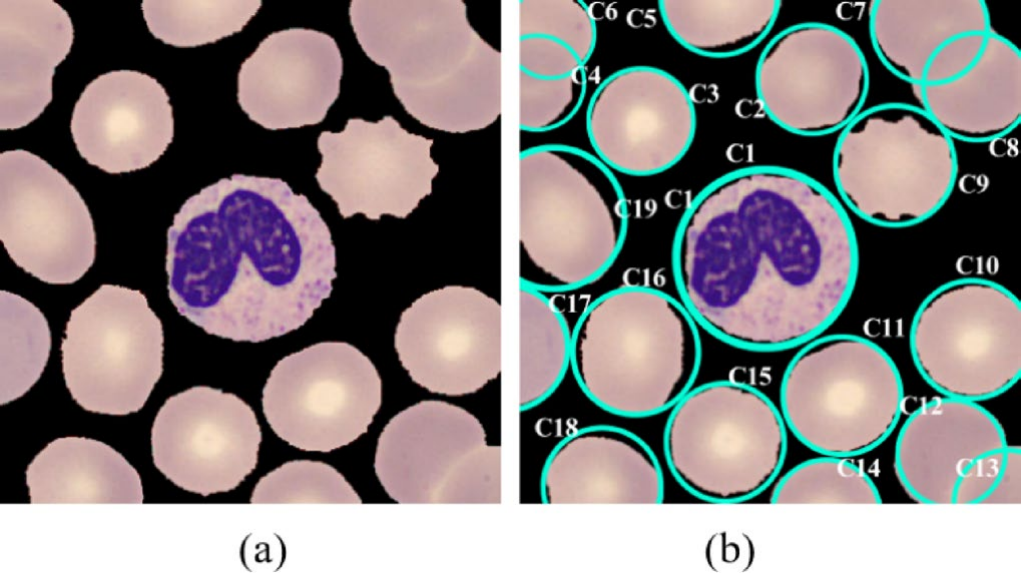
(**a**) Image before segmented by watershed algorithm, (**b**) after segmented by watershed algorithm.

In this study, we employed the watershed algorithm to detect and isolate individual blood cells from microscopic images. By utilizing morphological operations and distance transformation, the watershed algorithm delineated cell boundaries accurately. Subsequently, connected component analysis was performed to identify individual cells, and those with an area greater than a predefined threshold were saved as separate images for further analysis. This method ensured precise cell segmentation, vital for subsequent blood cell classification tasks. In Fig. 6, the image depicts the state before and after segmentation by the watershed algorithm. The working way of watershed algorithm is shown in algorithm 1. The study uses the Watershed algorithm for precise blood cell extraction as regions of interest (ROIs). To prevent over-segmentation, U-Net was first employed to create a clean binary mask, which is then refined using a marker-controlled watershed approach. This approach treats the segmented image as a topographic surface, with white pixels (blood cells) as peaks and black pixels (background) as valleys. The flooding process begins from markers placed at local minima, typically the centers of segmented regions, and expands outward. As the regions approach each other, boundaries are formed at the watershed line, accurately separating adjacent or overlapping cells. Morphological operations, such as erosion and dilation, are applied to refine the markers, further reducing over-segmentation and ensuring that segmentation occurs at the true boundaries of the cells. In our implementation, the U-Net mask was thresholded at τ = 0.5, then cleaned with a single morphological opening and closing using a 3 × 3 disk/box structuring element (1–2 iterations). The distance transform used the Euclidean (L2) metric with mask size 5; sure-foreground seeds were obtained by thresholding the normalized distance map at 0.30–0.35 of its maximum and enforcing a minimum inter-peak distance of 7 pixels (peak_local_max footprint 7 × 7) to avoid over-fragmentation. Sure-background was generated by dilating the binary mask three iterations (3 × 3 kernel). Markers were produced from 8-connectivity component labels; the watershed was run with compactness = 0.001 (skimage) / default (OpenCV), and ties were broken by gradient magnitude. Post-processing removed components with implausible size (area < 80 px or > 6,000 px), very low circularity (4πA/P²<0.40), or touching image borders; holes were filled once by binary closing. These settings were selected by a small grid search on the validation split to maximize mean IoU/Dice while visually minimizing over- and under-segmentation.

The Watershed algorithm treats the segmented image as a topographic surface, with white pixels (blood cells) as peaks and black pixels (background) as valleys. It simulates a flooding process, starting from markers placed at local minima (typically the centers of segmented regions) using distance transforms. As the flooding expands, boundaries are formed where regions approach each other, creating a watershed line at the local maxima. This ensures accurate separation of overlapping or connected cells, providing distinct boundaries even in areas where they were previously merged in the binary mask. In practice, we found the above thresholds and structuring-element sizes to be robust across folds; when images displayed dense overlap, increasing the min-distance to 9 pixels and lowering the DT threshold to 0.25 slightly reduced merges without inflating false splits, while all other parameters were kept fixed for reproducibility.

**Algorithm 1:**
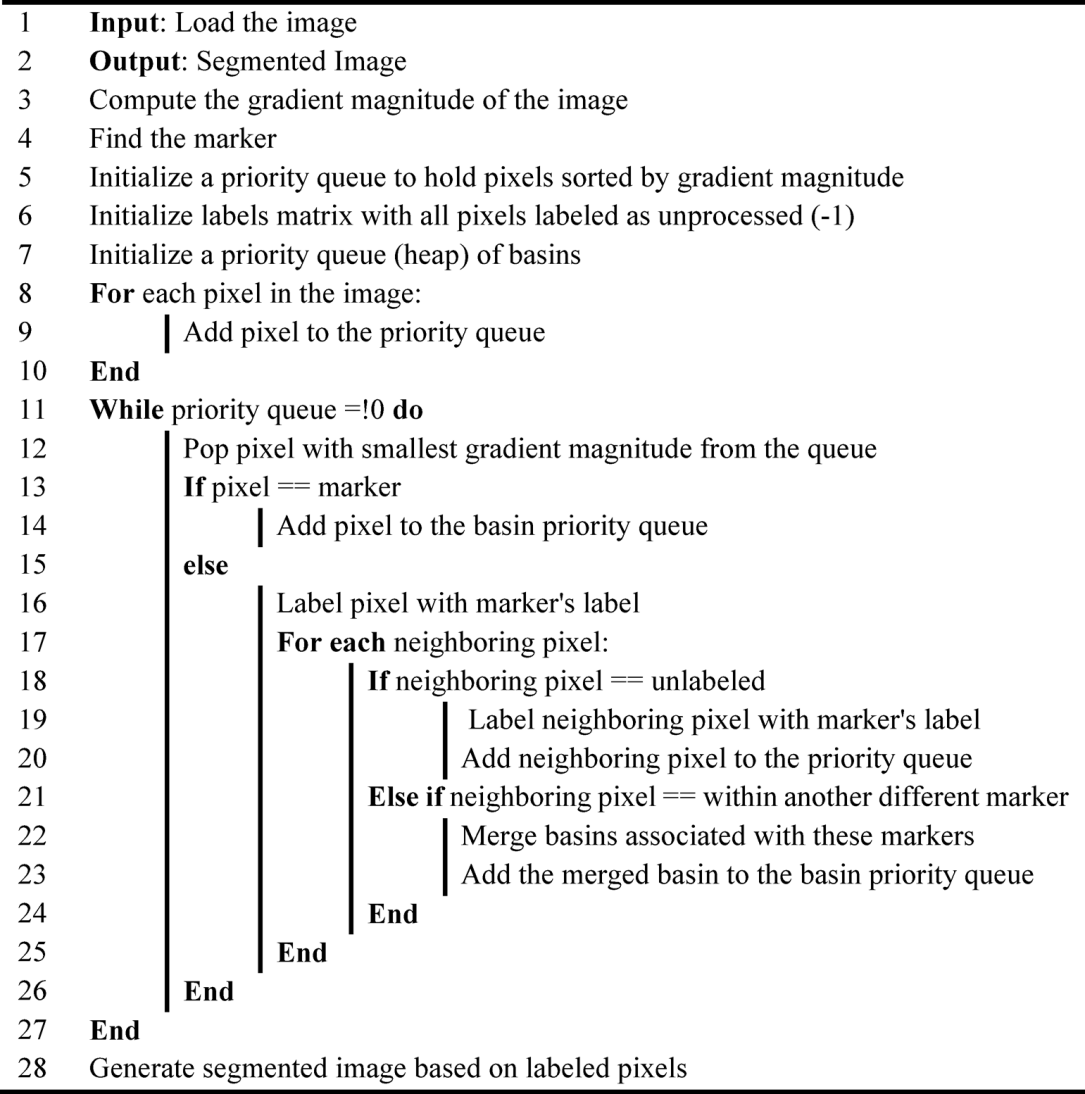
Watershed algorithm.

#### Data splitting

K-fold cross-validation is a technique used in machine learning to assess the performance of predictive models by partitioning the dataset into k equal-sized subsets. The model is trained on k-1 folds and evaluated on the remaining fold, with this process repeated k times. By averaging performance metrics across the folds, k-fold cross-validation provides an unbiased estimate of the model’s performance, reducing variance and enhancing robustness. K-fold cross-validation is an essential technique for reducing overfitting and improving generalization. By dividing the dataset into K subsets, the model is trained on K-1 folds and tested on the remaining one, ensuring that every data point is used for both training and validation. This iterative process provides a robust evaluation, reducing bias from a single train-test split and improving performance on unseen data. Additionally, it allows for effective parameter tuning, maximizes data utilization, and provides a comprehensive assessment of the model’s ability to generalize. This approach enhances the reliability and consistency of classification results, making it a valuable tool for evaluation and optimization.

**Fig. 7:**
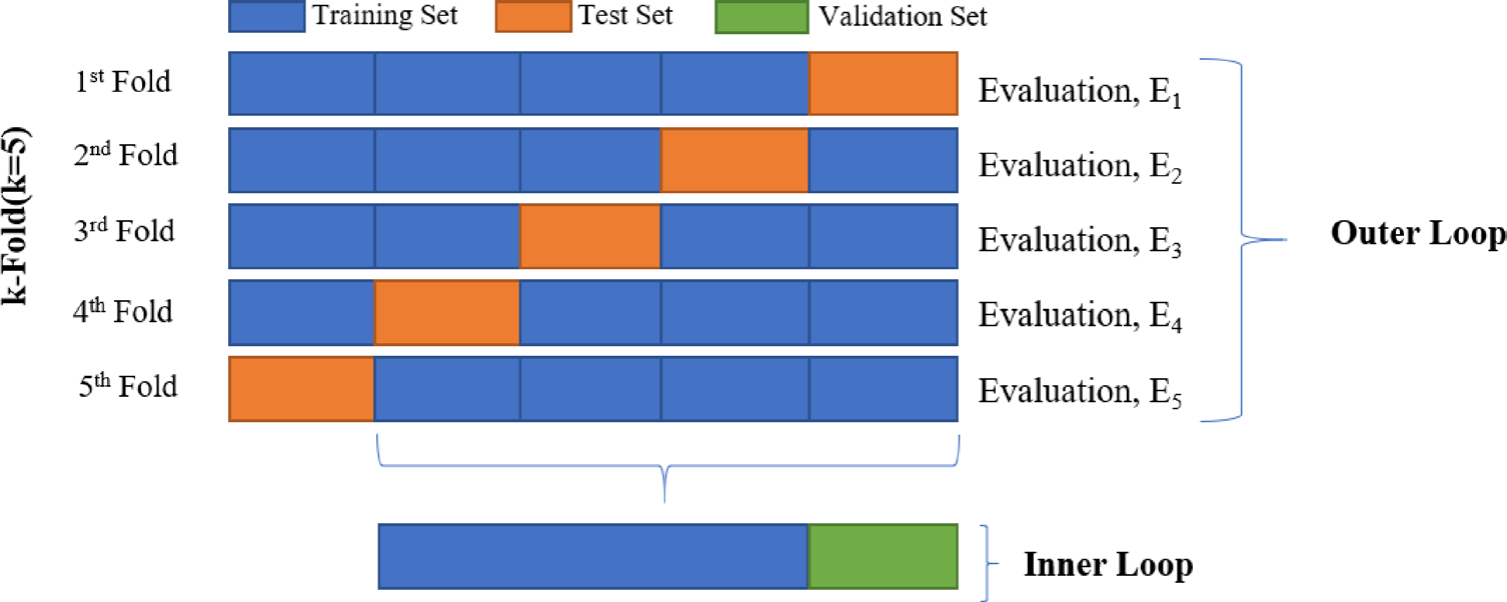
Data splitting technique of 5-fold cross validation technique.

In our study into blood cell classification utilizing the Mendeley repository dataset, we implemented a 5-fold cross-validation technique for computational efficiency and faster training. This entailed partitioning the dataset into five subsets, with four utilized for model training and the remaining one for testing in each iteration. This approach facilitated a thorough assessment of the model’s performance, maintaining a balanced utilization of the dataset. Figure 7 illustrates the data splitting mechanism employed for 5-fold cross-validation. The total number of images in each fold was 10,124, of which 6057 were for training, 2019 were for validation, and 2048 were for testing.

#### Proposed LWCNN model

**Fig. 8:**
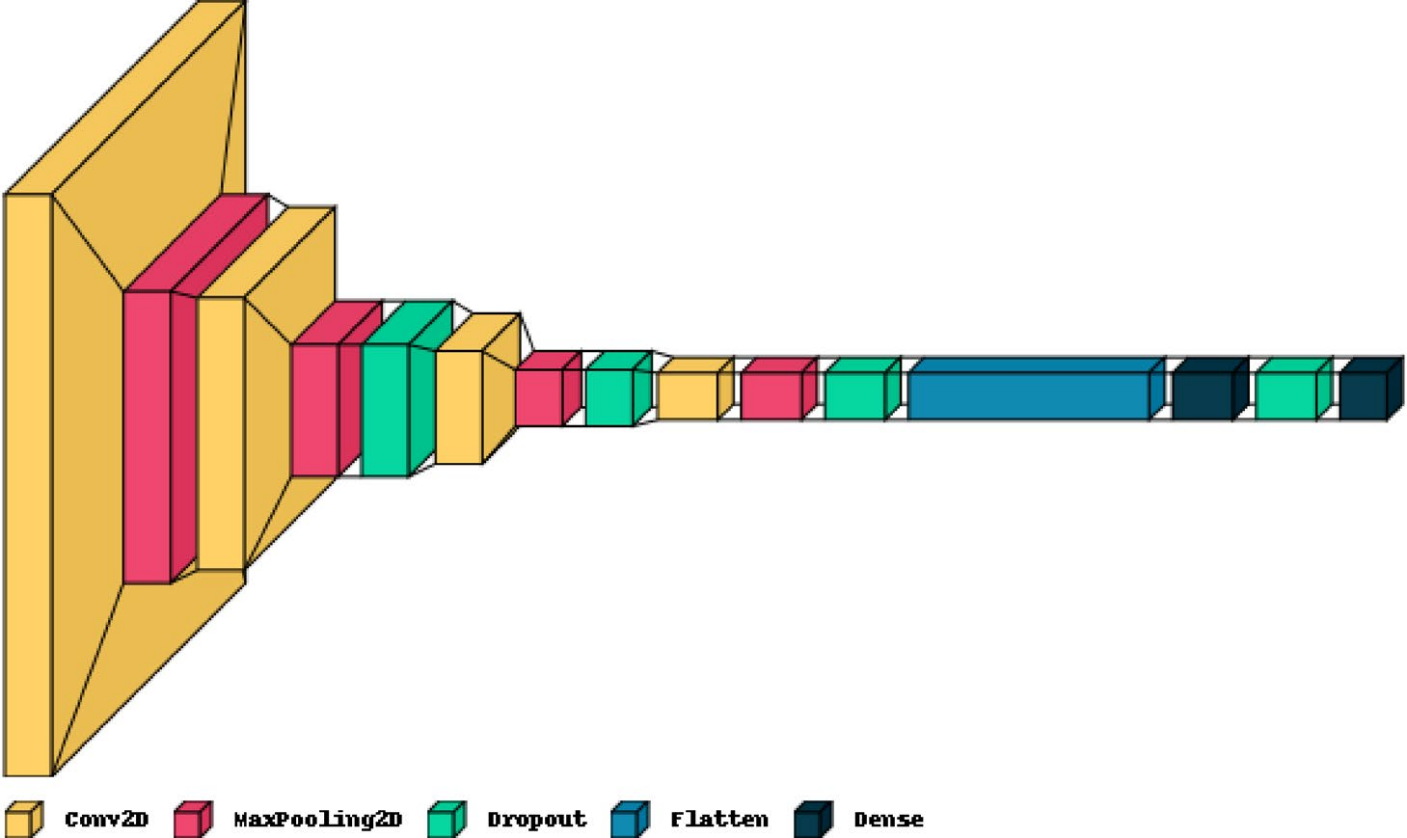
The proposed LWCNN architecture for blood cell classification. *(*Note: This figure has been depicted using the *visualkeras.layered_view()* function from the *VisualKeras Python package. * Source: https://github.com/paulgavrikov/visualkeras).

The proposed LWCNN model for blood cell classification exhibits a sophisticated architecture designed to effectively learn and distinguish features from input images. The model begins with a series of convolutional layers, each followed by max-pooling layers, which enable the extraction of hierarchical features while reducing spatial dimensions. Utilizing “ReLU” activation functions, these convolutional layers introduce non-linearity to the model, enhancing its capability to capture complex patterns. To prevent overfitting, dropout layers are strategically incorporated after each max-pooling operation, randomly dropping a fraction of the neuron units during training. This regularization technique helps to improve the model’s generalization performance by reducing the likelihood of learning noise or irrelevant features. The final layers of the model consist of densely connected layers, culminating in a “Softmax” activation function that produces probability distributions over the output classes. With a total of 653,129 trainable parameters, our LWCNN model demonstrates a considerable capacity to learn discriminative representations from the input blood cell images, paving the way for accurate classification results. Figure 8 shows the proposed LWCNN architecture where Table 4 provides a detailed overview of its structural components. Since the proposed model consists of only four convolutional layers, four max-pooling layers, and four dropout layers to reduce overfitting, as well as only 653,129 trainable parameters rather than millions of parameters of deep learning model, it is a lightweight CNN model that follows state of the art^84^. We adopt a LightWeight BloodCell-Net because the prior U-Net and marker-controlled watershed isolates single-cell ROIs, reducing classification to fine-grained cytomorphology. A four-stage 3 × 3 conv/2 × 2 max-pool stack provides sufficient receptive-field growth and invariance to smear variability while limiting capacity to curb overfitting on modest ROIs. ReLU is retained for optimization stability and efficient inference; light post-pool dropout with augmentation controls variance without degrading minority-class sensitivity. A small dense head fuses mid-level texture and nuclear cues that depthwise-separable backbones may attenuate. In controlled comparisons (matched input and compute) with MobileNet-V2/-V3, ShuffleNet, and EfficientNet-Lite, these models offered no consistent accuracy gains and exhibited higher sensitivity to width multipliers and batch-norm tuning. Thus, a shallow CNN delivers comparable performance with lower tuning burden, better cross-fold stability, and simpler deployment for routine hematology workflows.

**Table 4:** Summary table of the proposed CNN architecture.

Layer (Type)	Feature Map	No. of Parameters	Kernel Size	Activation Function
Conv2D	(None, 62, 62, 32)	896	(3, 3)	ReLU
MaxPooling2D	(None, 31, 31, 32)	0	(2, 2)	-
Conv2D	(None, 29, 29, 64)	18,496	(3, 3)	ReLU
MaxPooling2D	(None, 14, 14, 64)	0	(2, 2)	-
Dropout	(None, 14, 14, 64)	0	-	-
Conv2D	(None, 12, 12, 128)	73,856	(3, 3)	ReLU
MaxPooling2D	(None, 6, 6, 128)	0	(2, 2)	-
Dropout	(None, 6, 6, 128)	0	-	-
Conv2D	(None, 4, 4, 256)	295,168	(3, 3)	ReLU
MaxPooling2D	(None, 2, 2, 256)	0	(2, 2)	-
Dropout	(None, 2, 2, 256)	0	-	-
Flatten	(None, 1024)	0	-	-
Dense	(None, 256)	262,400	-	ReLU
Dropout	(None, 256)	0	-	-
Dense	(None, 9)	2,313	-	Softmax

The main novelty of the BloodCell-Net approach lies in its ability to efficiently and robustly classify nine types of blood cells, including erythrocytes, despite their similar texture and color. The model demonstrates high robustness through K-fold validation, using 6,057 training samples and 2,048 test samples across different folds. It is also lightweight, incorporating minimal convolutional layers and fewer trainable parameters. Additionally, the approach enhances classification accuracy by extracting key blood cell regions while removing noise and unwanted areas through a hybrid U-Net and Watershed segmentation algorithm. Furthermore, Contrast Limited Adaptive Histogram Equalization (CLAHE) and proper data augmentation improve image quality and diversity, making the model more effective in real-world applications.

## Result and discussion

**Fig. 9:**
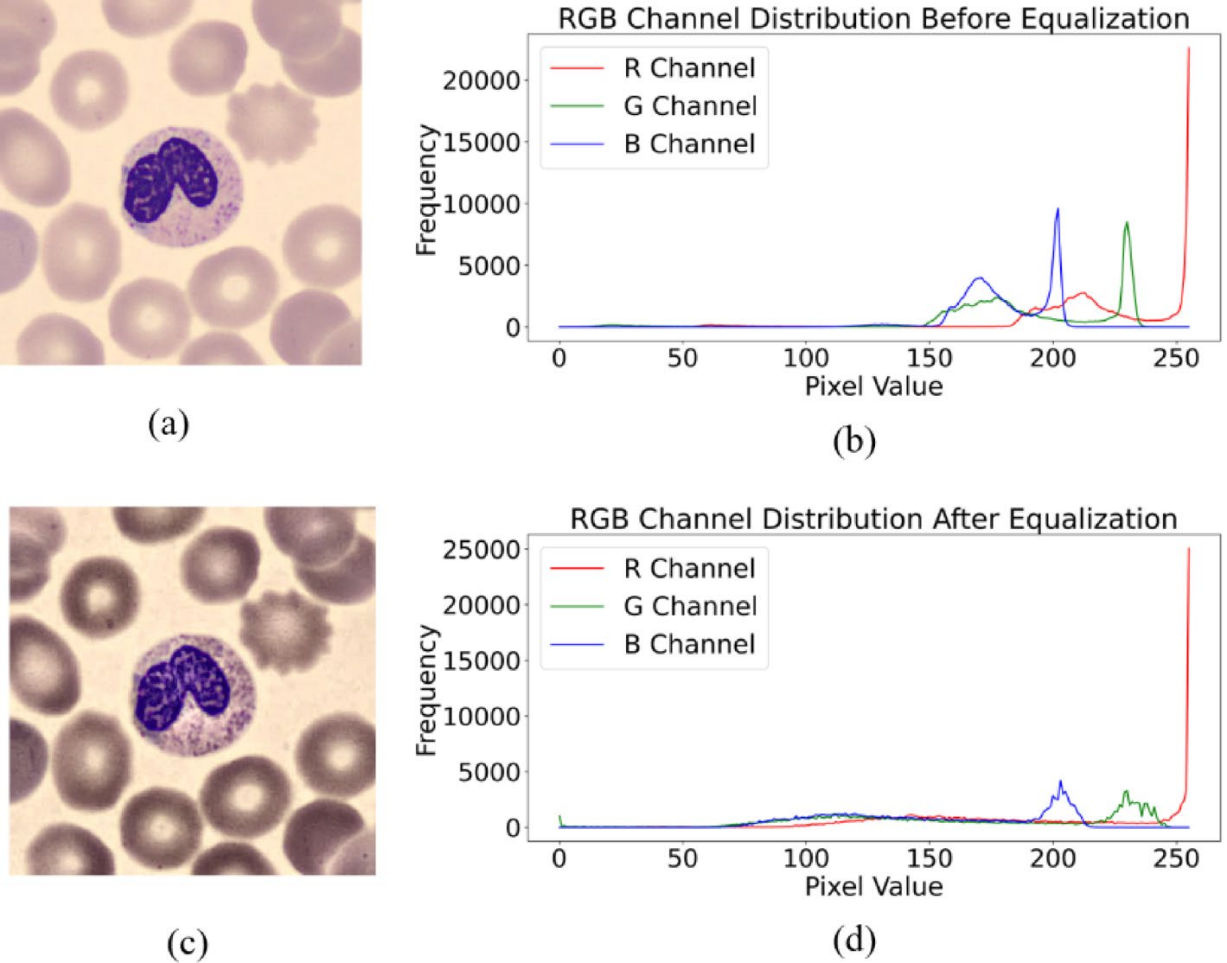
(**a**) Original image, (**b**) RGB channel distribution before applying CLAHE, (**c**) Histogram equalized image, (**d**) RGB channel distribution after applying CLAHE.

CLAHE is applied to enhance the contrast of our dataset images, effectively highlighting the distinguishing features between subjects and background. This enhancement plays a pivotal role in enabling the U-Net model to accurately segment blood cells amidst noisy backgrounds. A randomly selected image sample from our dataset is displayed in Fig. 9 alongside its histogram-equalized version. The RGB channel distribution is also depicted, illustrating that the image’s RGB channel distribution exhibits sharp peaks prior to histogram equalization, while the channels become more uniform after applying CLAHE.

Several evaluation metrics are used, each focusing on a different component of our proposed model’s performance, thereby assessing how well it classifies the blood cell.

### Accuracy

Accuracy indicates how well the model’s predictions correspond with the actual outcomes. It basically evaluates the overall performance of the model. It is computed with the following formula.

9 $$\:Accuracy=\frac{TP+TN}{TP+TN+FP+FN}$$

### Precision

This assesses how well the model can lower false positives, demonstrating the accuracy with which types of blood cell cases are confirmed. It is described as.

10 $$\:Precision=\frac{TP}{TP+FP}$$

### Recall

Recall presents the capacity of the model to detect every instance of a real blood cell and is as follows.

11 $$\:Recall=\frac{TP}{TP+FN}$$

### F1 score

The trade-offs between recall and precision are balanced by the F1-Score. Calculated as the harmonic mean of these two measures, it equals-.

12 $$\:F1=2\times\:\frac{Precision\times\:Recall}{Precision+Recall}$$

Where, TP = True Positive; TN = True Negative; FP = False Positive; FN = False Negative.

To address the inherent challenges such as similar color and texture in erythrocytes, we utilized U-Net, a robust deep learning architecture, to segment blood cells from microscopic images. U-Net excels in feature extraction and precise boundary delineation, which is crucial when dealing with the subtle differences between erythrocytes and other cell types. The model generates a binary mask, where the background is removed, and the regions of interest (ROIs) are clearly highlighted, effectively isolating the blood cells from complex and noisy elements in the smear.

In addition to U-Net, we incorporated the Watershed algorithm as a post-processing step to refine segmentation. Given the presence of overlapping erythrocytes, Watershed is particularly useful in separating touching cells by treating the binary mask as a topographic surface. This ensures precise delineation of individual cells, enhancing segmentation accuracy. The combined approach of U-Net and Watershed enables robust and reliable blood cell segmentation, crucial for further analysis and diagnosis.

Pixel-level and object-level metrics are crucial for evaluating the performance of segmentation models like U-Net. Pixel accuracy, precision, and sensitivity are key pixel-level metrics, while Intersection over Union (IoU) and the Dice coefficient assess segmentation quality at the object level.

Pixel accuracy measures the proportion of correctly segmented pixels out of the total pixels in the image. The model achieved 98.23%-pixel accuracy, indicating highly precise segmentation across the dataset. Precision, which quantifies the accuracy of positive predictions, reached 98.40%, demonstrating the model’s ability to minimize false positives and ensure that detected objects are indeed blood cells. Sensitivity (recall) was 98.26%, reflecting the model’s robustness in correctly identifying most of the actual blood cell pixels, reducing the likelihood of missing relevant structures.

At the object level, the 95.97% IoU score highlights the strong agreement between the predicted and actual blood cell regions, ensuring minimal overlap errors. The 97.92% Dice coefficient further confirms the high segmentation precision by indicating a near-perfect overlap between the predicted and ground truth masks. These high metric values emphasize the reliability of U-Net in effectively distinguishing blood cells from the background while maintaining precise boundaries.

The strong performance across both pixel-level and object-level metrics underscores U-Net’s effectiveness in handling complex blood smear images, where accurate delineation is critical for downstream analysis. Table 5 presents a detailed breakdown of these performance metrics.

**Table 5:** Performance metrics for U-Net model, including pixel-level and object-level evaluations.

Run	Accuracy	Precision	Sensitivity	IOU	Dice Coefficient
1	98.45	98.58	98.37	96.22	98.03
2	98.19	98.33	98.23	95.90	97.95
3	98.22	98.40	98.12	95.97	97.91
4	98.17	98.32	98.18	95.85	97.78
5	98.12	98.35	98.38	95.92	97.93
Mean ± Std	98.23 ± 0.12	98.40 ± 0.09	98.26 ± 0.10	95.97 ± 0.13	97.92 ± 0.08

**Fig. 10:**
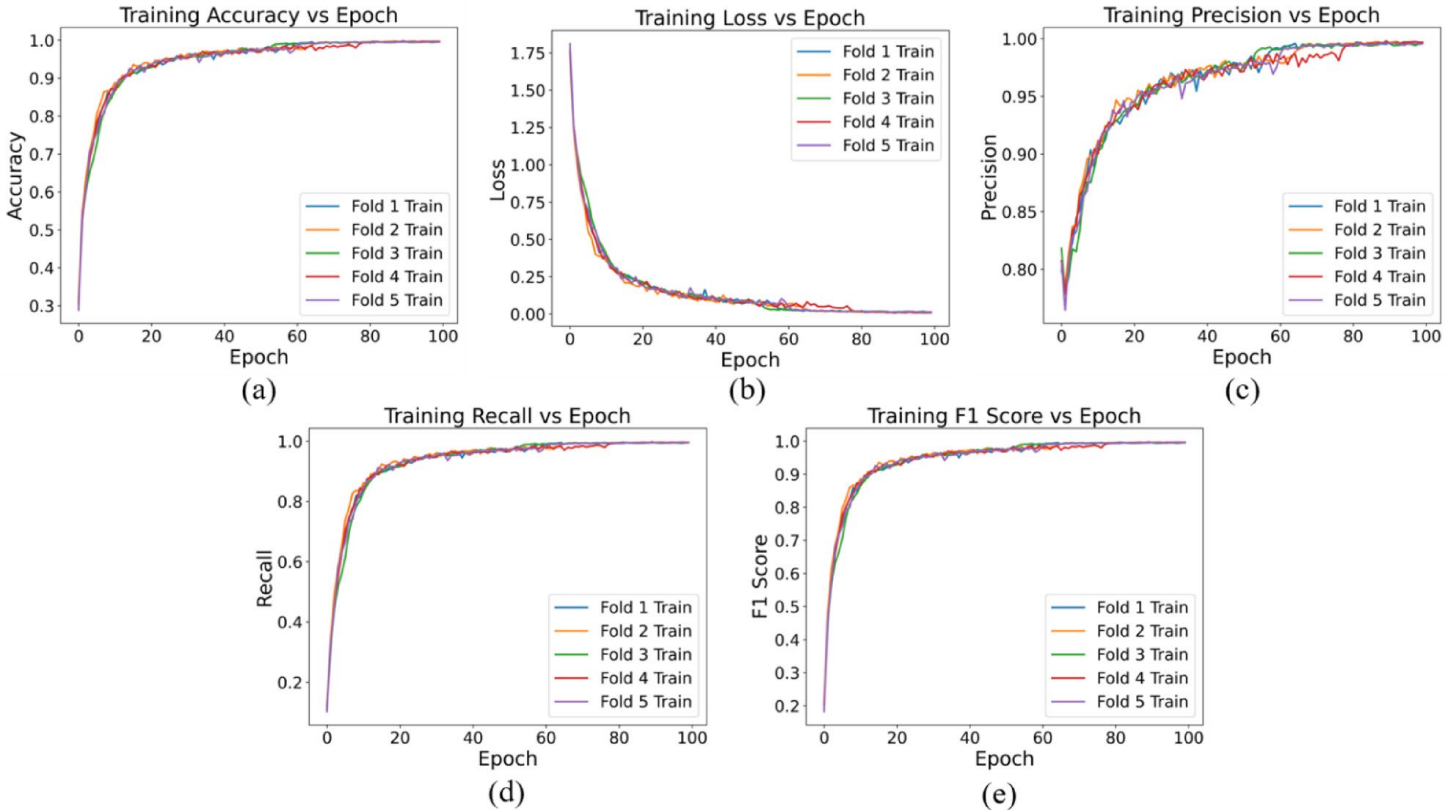
Training profile of the proposed LWCNN model, (**a**) Accuracy vs. epoch, (**b**) Loss vs. epoch, (**c**) Precision vs. epoch, (**d**) Recall vs. epoch, (**e**) F1 Score vs. epoch.

**Fig. 11:**
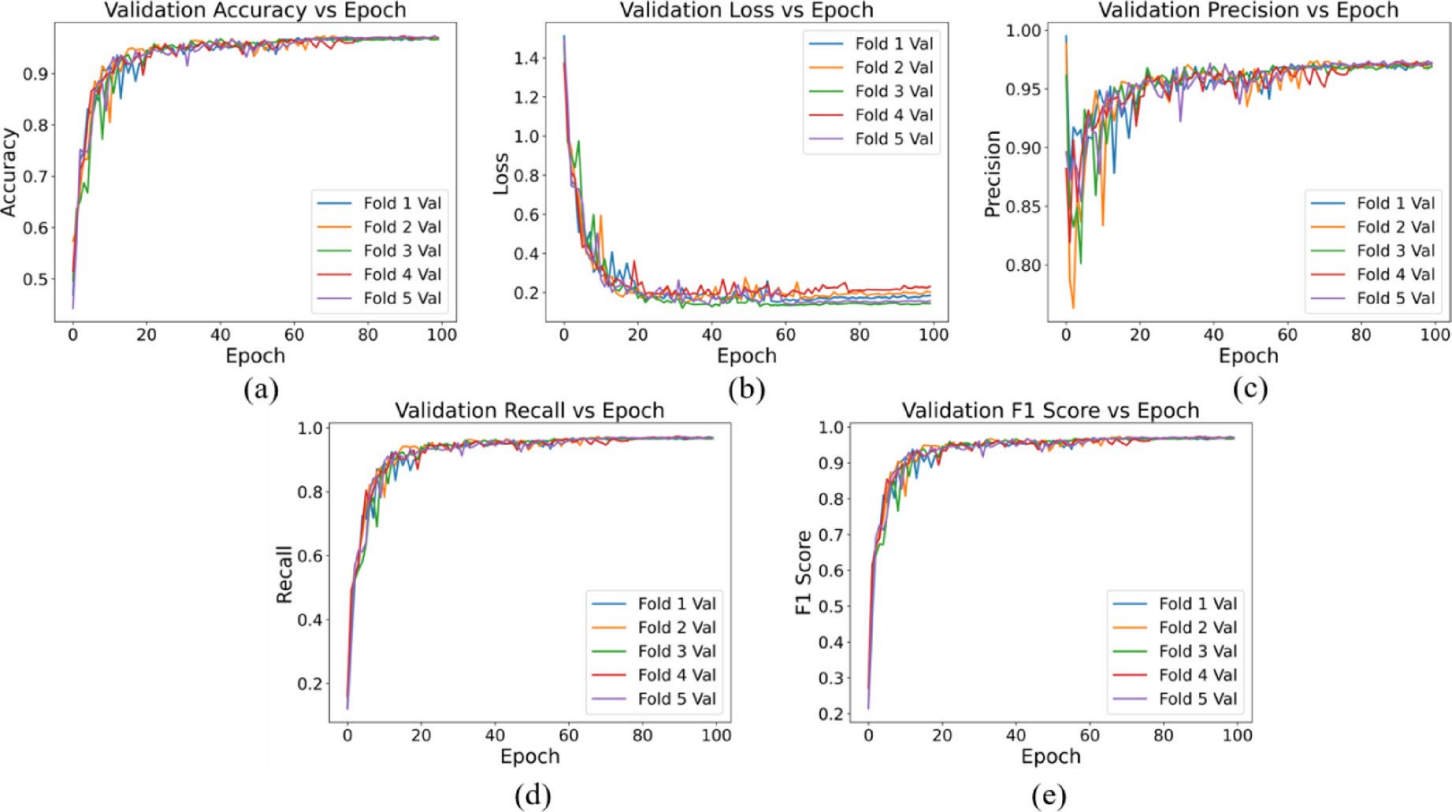
Validation profile of the LWCNN model, (**a**) Accuracy vs. epoch, (**b**) Loss vs. epoch, (**c**) Precision vs. epoch, (**d**) Recall vs. epoch, (**e**) F1 Score vs. epoch.

Our CNN model was trained specifically on single blood cells extracted from comprehensive microscopic blood cell images utilizing the watershed algorithm. The dataset comprised a total of 10,614 images distributed across nine distinct classes. Figures 10 and 11 present an overview of the model’s performance throughout the training process across all five-fold data. The training and validation accuracy versus epoch curve serve as an indicator of the model’s learning efficacy, with ascending accuracy suggesting iterative improvement. Meanwhile, the loss versus epoch curve monitors the model’s error minimization trajectory during training, with a decreasing trend indicative of effective learning while mitigating overfitting risks. Precision versus epoch and recall versus epoch curves delineate the model’s proficiency in accurately identifying positive samples and capturing all relevant positive instances, respectively, both pivotal in domain-specific performance assessments. Lastly, the F1 score versus epoch curve amalgamates precision and recall metrics, furnishing a balanced assessment of model performance. Continuous monitoring of these metrics facilitates a nuanced understanding of model behavior, thereby guiding strategic refinements to architecture, hyperparameters, and training protocols to optimize overall performance and generalization capabilities.

**Fig. 12:**
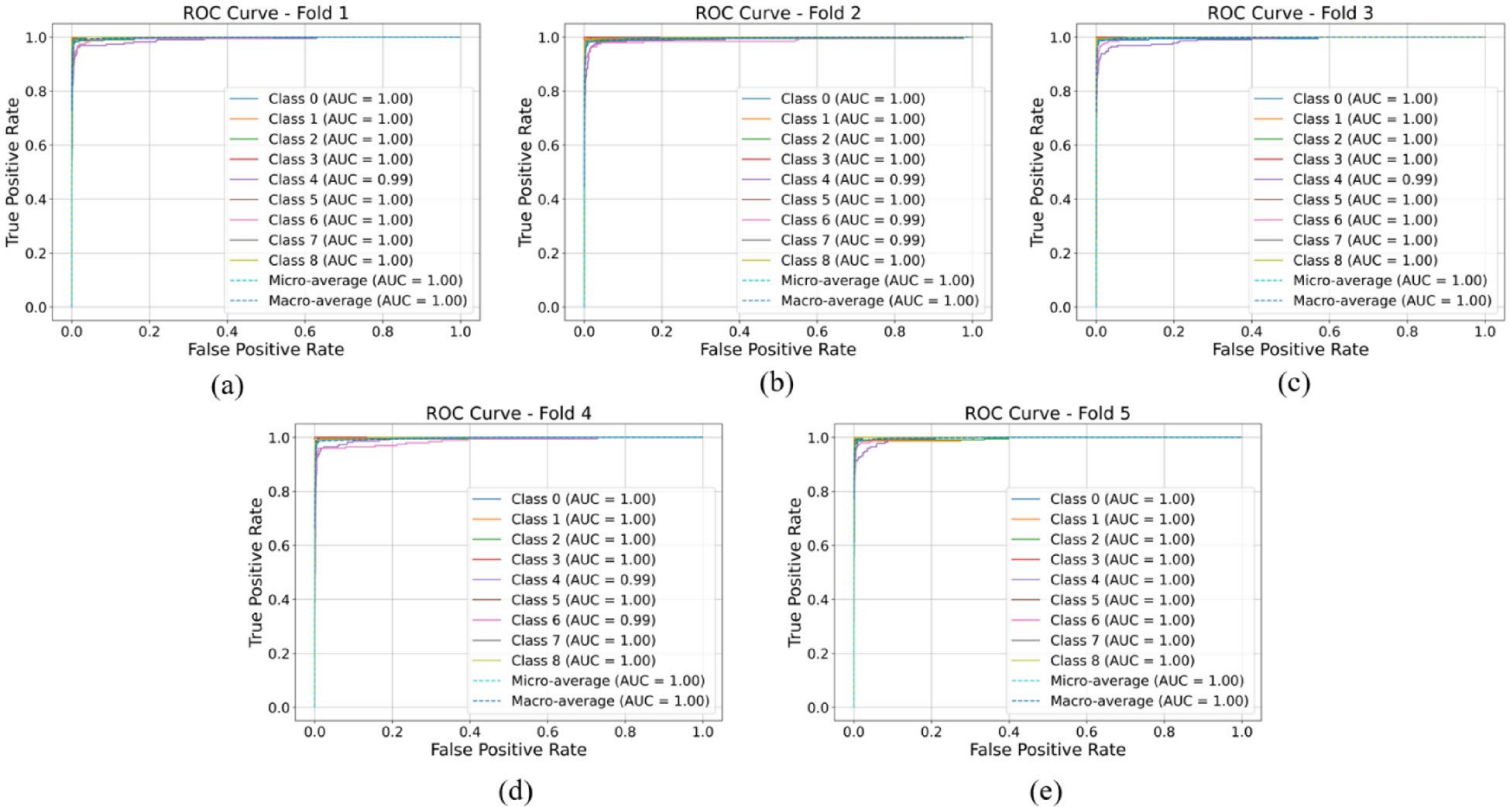
Receiver operating characteristic curve for (**a**) Fold-1, (**b**) Fold-2, (**c**) Fold-3, (**d**) Fold-4 and (**e**) Fold-5.

In a classifiers model, ROC curves offer valuable insights into the performance of a CNN model during training. These curves illustrate the model’s ability to discriminate between different types of blood cells, showcasing the trade-off between sensitivity (the ability to correctly identify positive instances) and specificity (the ability to correctly identify negative instances) at various threshold settings. Analyzing the ROC curve allows us to gauge how well the CNN model distinguishes between different blood cell types, with curves that hug the upper-left corner indicating superior performance. This understanding guides the selection of decision thresholds tailored to the specific requirements of blood cell classification tasks, such as prioritizing sensitivity to minimize misclassification of diseased cells or prioritizing specificity to minimize misclassification of healthy cells. Moreover, the area under the ROC curve (AUC) provides a comprehensive measure of the CNN model’s overall discriminatory ability across all threshold settings, facilitating informed decision-making in blood cell classification applications. Figure 12 displays the ROC curves for all five-fold data along with the corresponding AUC values for each class, as well as the micro-average AUC and macro-average AUC.

The performance of a CNN classifier can be evaluated using several evaluation metrics, including accuracy, precision, recall, and F1 score. Accuracy measures the proportion of correctly classified instances out of the total number of instances. However, in medical image analysis, precision and recall are particularly crucial metrics. Precision represents the ratio of correctly identified positive cases to all cases classified as positive, emphasizing the model’s ability to avoid false positives. Recall, on the other hand, measures the ratio of correctly identified positive cases to all actual positive cases, highlighting the model’s capability to capture all relevant instances of a particular class, thus minimizing false negatives. For instance, in medical image analysis, where the consequences of missing a diagnosis (false negative) or making an incorrect diagnosis (false positive) can be severe, the F1 score provides a comprehensive assessment of the classifier’s performance. A high F1 score indicates that the classifier has achieved both high precision and high recall, striking an optimal balance between minimizing false positives and false negatives. Table 6 presents the performance metrics of our proposed CNN model across all folds and their respective averages. It is noteworthy that the model achieved an average accuracy of 97.10%, precision of 97.19%, recall of 97.01%, and F1 score of 97.10%.

**Table 6:** Performance metrics for the proposed CNN classifier model for 5-fold cross validation.

Fold Number	Accuracy (%)	Precision (%)	Recall (%)	F1 Score (%)
Fold-1	97.07	97.11	96.92	97.01
Fold-2	97.41	97.45	97.31	97.37
Fold-3	96.72	96.87	96.72	96.79
Fold-4	97.46	97.60	97.41	97.50
Fold-5	96.82	96.96	96.72	96.83
Average	97.10	97.19	97.01	97.10

**Fig. 13:**
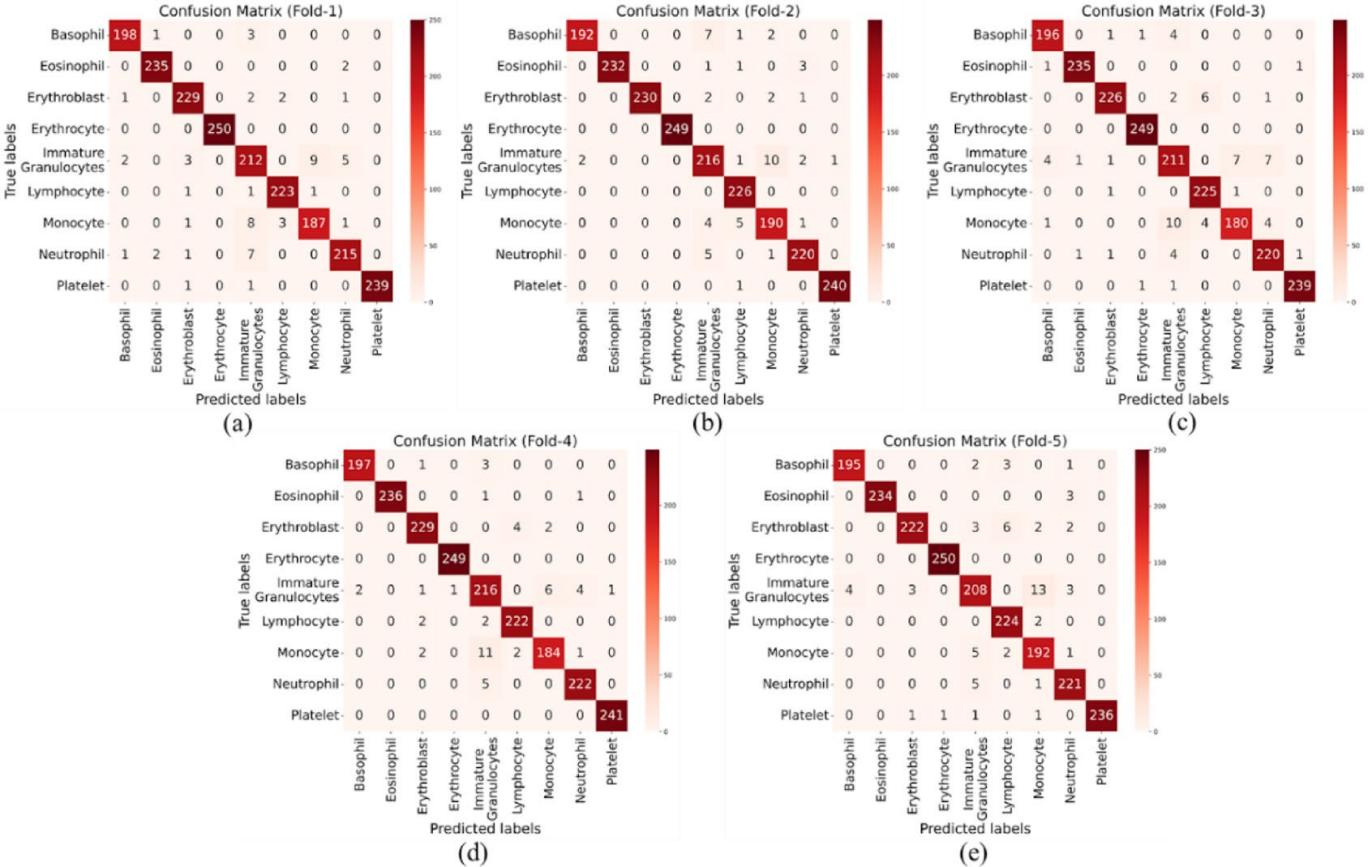
Confusion matrix of the LWCNN model on test set for (**a**) Fold-1, (**b**) Fold-2, (**c**) Fold-3, (**d**) Fold-4, (**e**) Fold-5.

The confusion matrix serves as a valuable tool for assessing the model’s performance by providing a detailed breakdown of predicted versus actual classifications for each class. Each row in the confusion matrix corresponds to the actual class, while each column represents the predicted class. For example, if we consider the class “Basophil” as an actual class and “Eosinophil” as a predicted class, the corresponding cell in the confusion matrix would indicate the number of instances where a Basophil was incorrectly classified as an Eosinophil. In Fig. 13, the confusion matrices for all folds of data are presented.

**Fig. 14:**
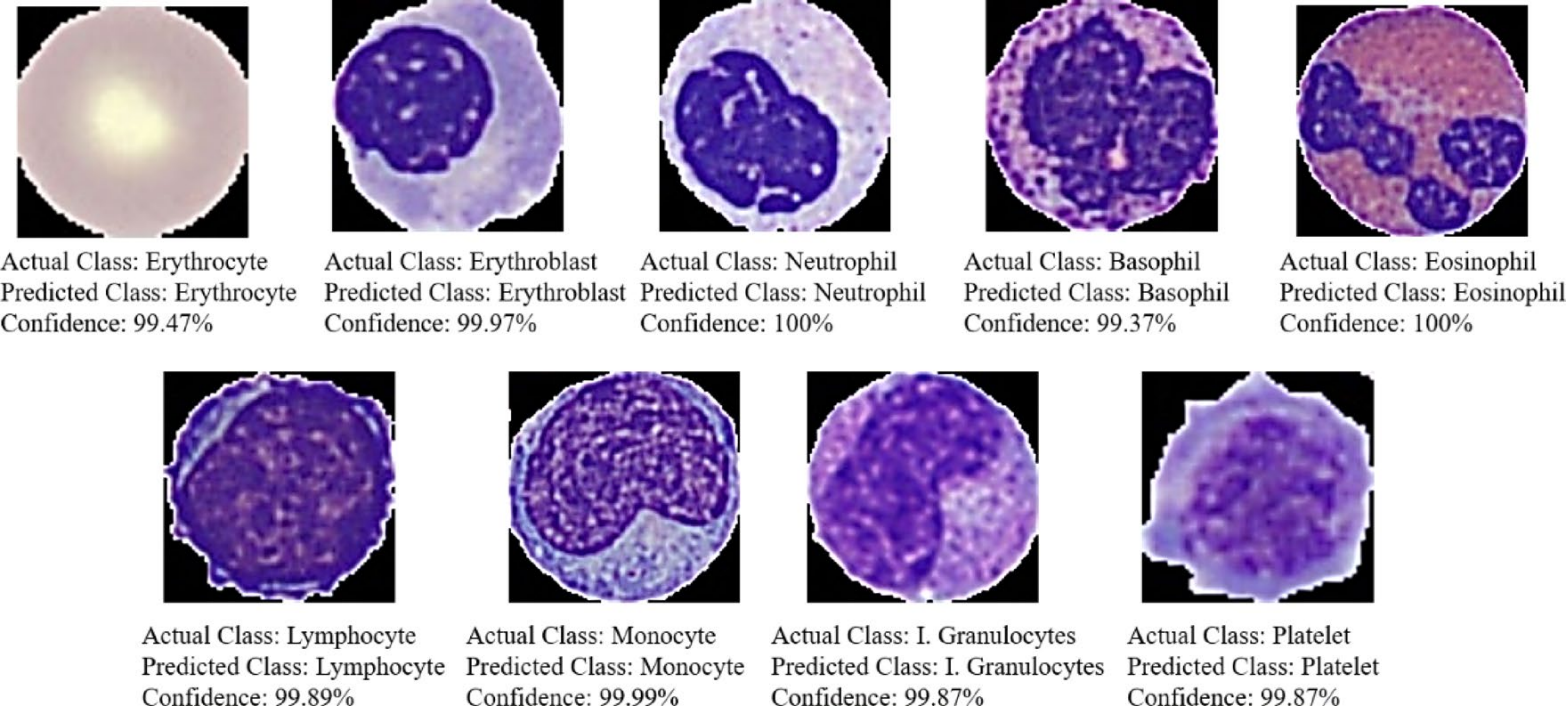
A representative subset of the test dataset, showcasing the confidence scores assigned by the classifier model for classification purposes.

A compact portion of the test dataset is depicted in Fig. 14, detailing the actual class, predicted class, and the confidence score provided by the model. Notably, all cells are correctly identified with over 99% confidence by the model.

The performance of a CNN classifier is typically evaluated using various metrics, including accuracy, precision, recall, and F1 score. Accuracy measures the proportion of correctly classified instances, but in medical image analysis, precision and recall are more critical due to the high stakes of incorrect diagnoses. Precision indicates the ratio of correctly identified positive cases to all cases classified as positive, reflecting the model’s ability to avoid false positives. Recall, conversely, measures the ratio of correctly identified positive cases to all actual positive cases, emphasizing the model’s ability to capture all relevant instances, thus minimizing false negatives. In medical contexts, where both false negatives and false positives can have severe consequences, the F1 score is a crucial metric as it combines precision and recall to provide a balanced evaluation of the model’s performance.

In our study, we evaluated the performance of the BloodCell-Net model using these metrics. As shown in Table 6, the model achieved an average accuracy of 97.10%, precision of 97.19%, recall of 97.01%, and F1 score of 97.10%. These results demonstrate the model’s ability to classify blood cells with high precision while minimizing both false positives and false negatives. Compared to state-of-the-art models in Table 7, our approach, which focuses on classifying nine blood cell types, performs competitively with accuracy levels similar to more complex models like DenseNet 161 and Hybrid methods (which reach accuracy above 99% but focus on fewer classes). Our model’s advantage lies in its lightweight architecture—with only 653,129 trainable parameters—making it faster to train and more computationally efficient, while still delivering competitive classification performance.

**Table 7:** Comparative analysis with state-of-the-art models in blood cell classification.

Ref.	Classifier	Dataset	Accuracy
^78^	AlexNet, VGG Net 13, 11, ResNet 18, 34, 50, SqueezeNet 10, 11, DenseNet 121, 161.	BCCD dataset (four classes)	100% (DenseNet 161)
^79^	DT, RF, SVM, k-NN, CNN, VGG16, ResNet15	IEEE data port (3539images) Five classes	97% (RF)
^80^	CNN, ELM, AlexNet, GoogleNet, VGG-16, and ResNet	BCCD dataset (four classes)	96.03%
^81^	CNN	Kaggle	98.55%
^75^	Optimized CNN model	IEEE data port (3539images) (Five classes)	99.86%
^76^	CNN based MicrocellNet	Mendeley dataset (Eight classes)	97.65%
^77^	Hybrid method combination of the Inception module, pyramid pooling module (PPM), and depth wise squeeze-and-excitation block	1. Mendeley dataset (Eight classes) 2. IEEE data port (3539images) (Five classes) 3. BCCD dataset (four classes)	1. 99.72% 2. 99.22%, 3. 99.96%
^77^	Multibranch lightweight CNN (IM + DSE + PPM)	BCCD (Four classes)	99.96%
^85^	Efficient Model (EMO-6 M) with knowledge distillation	Single-cell PB smear dataset (11,261 images) Six classes	93.55%
^86^	Efficient lightweight CNN (LW_CNN)	Custom Dataset (4 Classes)	100%
**Our Proposed**	**BloodCell-Net Approach** **(LWCNN)**	Preprocessed Blood cells (**9 classes**,** including erythrocytes**) Source: Mendeley dataset	**97.10%**

**Fig. 15:**
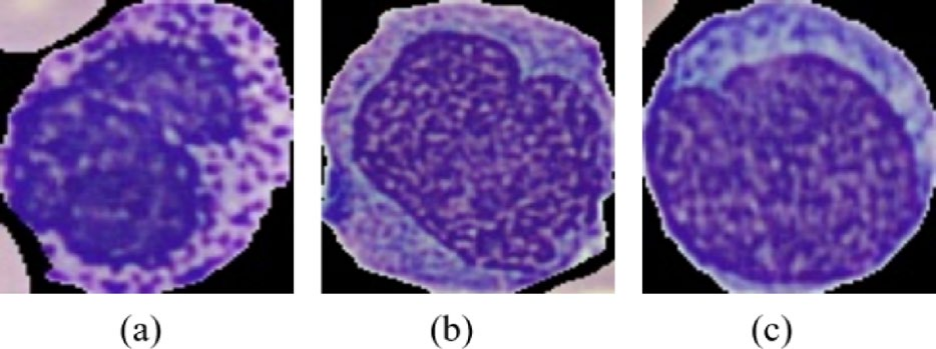
The blood cell images that were misclassified by our model: (**a**) Basophil as Immature Granulocyte, (**b**) Monocyte as Immature Granulocyte, and (**c**) Monocyte as Lymphocyte.

By examining the diagonal elements of the confusion matrix, which represent correct classifications, and off-diagonal elements, which represent misclassifications, we can better understand where the model struggles. From the analysis, we observe that Monocyte cells are sometimes misclassified as Immature Granulocytes, Monocytes as Lymphocytes, and Basophils and Immature Granulocytes are also confused. These misclassifications suggest that the model faces certain challenges in distinguishing these classes. Several factors contribute to these misclassifications. First, low-quality images, such as those with poor resolution, blurred edges, or inconsistent contrast, can make it difficult for the model to distinguish between similar cell types. This is particularly problematic for cell types like Monocytes and Immature Granulocytes, or Lymphocytes and Monocytes, which can appear very similar under such conditions. Additionally, the model may place too much emphasis on color differences, even though color is not always the most reliable feature for distinguishing between cell types. For example, Monocytes might be confused with Lymphocytes or Immature Granulocytes when their color tones appear similar or inconsistent across images, influenced by lighting conditions. Figure 15 presents some wrongly classified images by the CNN model.

Furthermore, the shapes and structures of certain cells, such as Monocytes, Immature Granulocytes, and Lymphocytes, can be difficult to differentiate, as they may share similar rounded appearances with subtle differences in internal structures. Misclassification can also arise from inaccurate labeling of the dataset, where a Monocyte might be mislabeled as an Immature Granulocyte, leading to confusion during training.

**Fig. 16:**
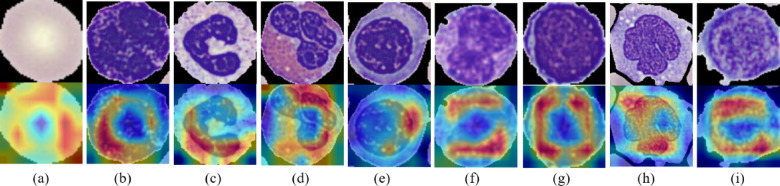
Class-wise Grad-CAM visualization of blood cell; (**a**) Erythrocyte; (**b**) Basophil; (**c**) Neutrophil; (**d**) Eosinophil; (**e**) Erythroblast; (**f**) Immature Granulocytes; (**g**) Lymphocyte; (**h**) Monocyte; (**i**) Platelet.

Finally, an explainable AI approach incorporated with the Grad-CAM technique has been employed to visualize the region with the most significant influence in classifying blood cells. Figure 16 illustrates how the output of the Grad CAM module in our model was transparent to the prediction of each class of blood cell. The grad map in Fig. 16 shows the places the model pays the most attention to during classification, such as warmer colours (like red and yellow), while the more fabulous colours (like blue) areas receive the least attention. The centre of the maps is where we find the salient areas, indicating that the model leverages features extracted from places in the input blood cell images and makes the appropriate classification inferences.

Additionally, the BloodCell-Net model effectively identified the nine types of blood cells by emphasizing their distinct morphologic and staining features (like red and yellow), resembling expert hematological diagnosis. Figure 16 illustrates that in erythrocytes, the model brought into focus the mid-pale area (red) related to their biconcave shape and plays a critical role in their distinction from the nucleated cells. In basophils, the attention of the model (red and yellow) has been drawn to the dense basophilic granules within the cytoplasm, a key identifier that frequently darkens the core due to intense staining. Neutrophils were recognized by the highlighted multi-lobed nucleus and fine cytoplasmic granules, features essential for differentiating them from immature granulocytes. The model identified eosinophils through strong attention to their large, defining eosinophilic (red-orange) granules, which contain major basic proteins and are critical for accurate recognition. For erythroblasts, the heatmap emphasized both the round nucleus and deeply basophilic cytoplasm, typical traits of nucleated immature red cells that help distinguish them from other lymphoid cells. The model identified immature granulocytes through emphasis on the unsegmented nucleus and immature cytoplasm, which are characteristic of granulocytic precursors rather than of mature neutrophils. The Grad-CAM identified lymphocytes by their typical large and round nucleus with a little cytoplasm in both peripheral blood smears. The model attended to the kidney-shaped nucleus and abundant pale-gray cytoplasm of monocytes, features routinely used by experts to distinguish them from lymphocytes and neutrophils. Finally, even for platelets, despite their small size, the model effectively focused on granule-rich regions and shape boundaries, enabling it to accurately distinguish true thrombocytes from background noise or cellular debris. The analysis indicates that Grad Camp validates the BloodCell-Net model in the extraction of meaningful, expert-recognized regions as well as features for blood cell classification.

Table 8 presents an ablation/sensitivity analysis quantifying each component’s contribution. The full pipeline (CLAHE + augmentation + U-Net + watershed) attains 97.10% accuracy and 97.01% recall. Removing CLAHE yields a modest decline (96.48% / 95.77%; −0.62/−1.24 pp), indicating contrast normalization is helpful but not dominant. Excluding augmentation causes a larger drop (95.08% / 95.02%; −2.02/−1.99 pp), underscoring its role in generalization. The largest degradation arises when bypassing ROI segmentation and training on direct tiles (85.57% / 83.58%; −11.53/−13.43 pp), confirming U-Net + watershed–based single-cell extraction is the principal driver of performance. Finally, adding two extra convolutional blocks does not recover accuracy (95.20% / 94.30%; −1.90/−2.71 pp) and increases computation, suggesting additional depth cannot compensate for missing/less informative preprocessing and tends to overfit modest ROIs. Collectively, these results justify our design: precise ROI extraction is indispensable, with augmentation and CLAHE providing complementary gains, while deeper backbones add cost without commensurate benefit.

**Table 8:** Ablation study: impact of preprocessing and architectural variants on BloodCell-Net performance.

Setting	Accuracy (%)	Sensitivity/Recall (%)	Notes
Full pipeline (CLAHE + Augmentation + U-Net + Watershed )	97.10	97.01	Proposed Pipeline
CLAHE	96.48	95.77	No CLEHA
Augmentation	95.08	95.02	No Augmentation
U-Net/Watershed (direct tiles)	85.57	83.58	No ROI segmentation
+ 2 extra conv blocks	95.20	94.30	Low accuracy, high computational time

## Conclusion

Classifying and counting blood cells are fundamental laboratory tests essential for hematologists to make informed medical decisions. However, the manual process of classifying and counting blood cells is time-consuming, prone to errors, and labor-intensive. To address this challenge, a deep learning-based blood cell classification BloodCell-Net framework has been proposed in the study. Several preprocessing steps, including image resizing, rescaling, histogram equalization, and data augmentation, are employed to enhance the performance of the segmentation model. A deep learning-based image segmentation technique called U-Net is utilized for segmenting the microscopic blood smear images to drop the complex and noisy background. In this research we utilized the standard U-Net architecture. Subsequently, a watershed algorithm is deployed to extract the single blood cells including overlapping cells. A custom-LWCNN model is proposed for classifying the single blood cells for counting. The proposed approach is the first study over the previous one that has included nine types of blood cells and classified them with the highest performance.

In the study, the lack of an annotated dataset made preparing the erythrocyte dataset challenging for the investigation. The over-segmentation of the watershed algorithm is another issue addressed using UNet incorporation. The study was conducted on limited data, which can be expanded incorporation with medical and diagnostic research centres. Reticulocytes and other blood cell subsets are still not considered in this study, but they can be carried out hereafter. Future research can be carried out on the deployment of the BloodCell-Net approach. Additionally, pruning, Bayesian, Particle Sawm optimization, and so on can be performed for parameter tuning and ensemble approach, and comprehensive clinical validation can also be incorporated. This study will open a new dimension for researchers in medical imaging.

## Data Availability

The dataset will be shared at the request of the corresponding author. Email: [simulhasantalukder@gmail.com](mailto: simulhasantalukder@gmail.com).

## References

[1] King, W., Toler, K. and Woodell-May, J., 2018. Role of white blood cells in blood-and bone marrow-based autologous therapies. BioMed research international, 2018. 10.1155/2018/6510842 PMC6077567 30112414

[2] Fathima, S. J. & Khanum, F. Blood Cells and Leukocyte Culture â€“A Short Review. *Open Access Blood Research & Transfusion Journal* **1**(2), 31–32 (2017).

[3] Liu, S. et al. Measurement of the refractive index of whole blood and its components for a continuous spectral region. *J. Biomed. Opt.* **24**(3), 035003–035003 (2019). 30848110 10.1117/1.JBO.24.3.035003 PMC6403469

[4] Zhou, P. et al. The associations between leukocyte, erythrocyte or platelet, and metabolic syndrome in different genders of Chinese. *Medicine* **95**(44), e5189 (2016). 27858856 10.1097/MD.0000000000005189 PMC5591104

[5] Badior, K. E. & Casey, J. R. Molecular mechanism for the red blood cell senescence clock. *IUBMB Life* **70**(1), 32–40 (2018). 29240292 10.1002/iub.1703

[6] Herron, C. Know your wbcs. *Nursing Made Incredibly Easy* **10**(1), 11–15 (2012).

[7] Russell, E. S. & Bernstein, S. E. Blood and blood formation. *Biology of the laboratory mouse* **2**, 351–372 (1966).

[8] Ramoser, H., Laurain, V., Bischof, H. and Ecker, R.. Leukocyte segmentation and classification in blood-smear images. In *2005 IEEE Engineering in Medicine and Biology 27th Annual Conference* (3371–3374). (IEEE, 2006) 10.1109/IEMBS.2005.1617200 17280945

[9] Mathur, A., Tripathi, A. S. & Kuse, M. Scalable system for classification of white blood cells from Leishman stained blood stain images. *Journal of pathology informatics* **4**(2), 15 (2013). 23766937 10.4103/2153-3539.109883 PMC3678750

[10] Habibzadeh, M., Krzyżak, A. & Fevens, T. Comparative study of shape, intensity and texture features and support vector machine for white blood cell classification. *Journal of Theoretical and Applied Computer Science* **7**(1), 20–35 (2013).

[11] Cengil, E., Çınar, A. & Yıldırım, M. A hybrid approach for efficient multi-classification of white blood cells based on transfer learning techniques and traditional machine learning methods. *Concurrency and Computation: Practice and Experience* **34**(6), e6756 (2022).

[12] Shafique, S. and Tehsin, S., 2018. Computer-aided diagnosis of acute lymphoblastic leukaemia. Computational and mathematical methods in medicine, 2018. 10.1155/2018/6125289 PMC5851334 29681996

[13] Xing, F. & Yang, L. Robust nucleus/cell detection and segmentation in digital pathology and microscopy images: a comprehensive review. *IEEE Rev. Biomed. Eng.* **9**, 234–263 (2016). 26742143 10.1109/RBME.2016.2515127 PMC5233461

[14] Gurcan, M. N. et al. Histopathological image analysis: A review. *IEEE Rev. Biomed. Eng.* **2**, 147–171 (2009). 20671804 10.1109/RBME.2009.2034865 PMC2910932

[15] Abbas, N., Mohamad, D., Abdullah, A.H., Saba, T., Al-Rodhaan, M. and Al-Dhelaan, A., 2015. Nuclei segmentation of leukocytes in blood smear digital images. Pakistan journal of pharmaceutical sciences, 28(5). 26408877

[16] Salem, N., Sobhy, N. M. & El Dosoky, M. A comparative study of white blood cells segmentation using otsu threshold and watershed transformation. *Journal of Biomedical Engineering and Medical Imaging* **3**(3), 15 (2016).

[17] Puttamadegowda, J. and Prasannakumar, S.C., White Blood cell sementation using Fuzzy C means and snake. In *2016 International Conference on Computation System and Information Technology for Sustainable Solutions (CSITSS)* (47–52). (IEEE, 2016)

[18] Marzuki, N. C., Mahmood, N. H. & Razak, M. A. Segmentation of white blood cell nucleus using active contour. *Jurnal teknologi* **74**(6), 115–118 (2015).

[19] Sharif, J.M., Miswan, M.F., Ngadi, M.A., Salam, M.S.H. and bin Abdul Jamil, M.M., February. Red blood cell segmentation using masking and watershed algorithm: A preliminary study. In *2012 international conference on biomedical engineering (ICoBE) *(258–262). (IEEE, 2012)

[20] Sadafi, Ario, Martin Radolko, Iosif Serafeimidis, and Steffen Hadlak. "Red blood cells segmentation: a fully convolutional network approach." *In 2018 IEEE Intl Conf on Parallel & Distributed Processing with Applications, Ubiquitous Computing & Communications, Big Data & Cloud Computing, Social Computing & Networking, Sustainable Computing & Communications (ISPA/IUCC/BDCloud/SocialCom/SustainCom)*, (911–914). (IEEE, 2018).

[21] Zhang, M., Li, X., Xu, M. and Li, Q.,. RBC semantic segmentation for sickle cell disease based on deformable U-Net. In *Medical Image Computing and Computer Assisted Intervention–MICCAI 2018: 21st International Conference, Granada, Spain, September 16–20, 2018, Proceedings, Part IV 11* (pp. 695–702). (Springer International Publishing, 2018)

[22] Ul Haq, I., Khan, M. T. & Sadique, U. An intelligent approach for blood cell detection employing faster RCNN. *Pakistan J. Eng. Technol.* **6**(1), 1–6 (2023).

[23] Abas, S. M., Abdulazeez, A. M. & Zeebaree, D. Q. A YOLO and convolutional neural network for the detection and classification of leukocytes in leukemia. *Indonesian Journal of Electrical Engineering and Computer Science* **25**(1), 200–213 (2022).

[24] Gautam, A. and Bhadauria, H. Classification of white blood cells based on morphological features. In *2014 International Conference on Advances in Computing, Communications and Informatics (ICACCI)* (2363–2368). (IEEE, 2014)

[25] Hegde, R. B., Prasad, K., Hebbar, H., Singh, B. M. K. & Sandhya, I. Automated decision support system for detection of leukemia from peripheral blood smear images. *J. Digit. Imaging* **33**, 361–374 (2020). 31728805 10.1007/s10278-019-00288-y PMC7165227

[26] Prinyakupt, J. & Pluempitiwiriyawej, C. Segmentation of white blood cells and comparison of cell morphology by linear and naïve Bayes classifiers. *Biomed. Eng. Online* **14**, 1–19 (2015). 26123131 10.1186/s12938-015-0037-1 PMC4485641

[27] Acevedo, A., Alférez, S., Merino, A., Puigví, L. & Rodellar, J. Recognition of peripheral blood cell images using convolutional neural networks. *Comput. Methods Programs Biomed.* **180**, 105020 (2019). 31425939 10.1016/j.cmpb.2019.105020

[28] Hegde, R. B., Prasad, K., Hebbar, H. & Singh, B. M. K. Feature extraction using traditional image processing and convolutional neural network methods to classify white blood cells: a study. *Australas. Phys. Eng. Sci. Med.* **42**, 627–638 (2019). 30830652 10.1007/s13246-019-00742-9

[29] Aliyu, H. A., Razak, M. A. A., Sudirman, R. & Ramli, N. A deep learning AlexNet model for classification of red blood cells in sickle cell anemia. *Int J Artif Intell* **9**(2), 221–228 (2020).

[30] Habibzadeh, M., Jannesari, M., Rezaei, Z., Baharvand, H. and Totonchi, M., 2018, April. Automatic white blood cell classification using pre-trained deep learning models: Resnet and inception. In: *Tenth international conference on machine vision (ICMV 2017)* (10696, 274–281). SPIE.

[31] Çınar, A. & Tuncer, S. A. Classification of lymphocytes, monocytes, eosinophils, and neutrophils on white blood cells using hybrid Alexnet-GoogleNet-SVM. *SN Applied Sciences* **3**, 1–11 (2021).

[32] Talukder, M. S. H., Sulaiman, R. B., Chowdhury, M. R., Nipun, M. S. & Islam, T. PotatoPestNet: a CTInceptionV3-RS-based neural network for accurate identification of potato pests. *Smart Agricultural Technol.* **5**, 100297 (2023).

[33] Allam, M. & Malaiyappan, N. Hybrid Feature Selection based on BTLBO and RNCA to Diagnose the Breast Cancer. *Int. Arab J. Inform. Technol.* **20**(5), 727–737 (2023).

[34] Talukder, M. S. H. & Akter, S. An improved ensemble model of hyper parameter tuned ML algorithms for fetal health prediction. *Int. J. Inf. Technol.* **16**(3), 1831–1840 (2024).

[35] Yu, X. et al. Deep learning for fast denoising filtering in ultrasound localization microscopy. *Phys. Med. Biol.* **68**(20), 205002. 10.1088/1361-6560/acf98f (2023). 10.1088/1361-6560/acf98f 37703894

[36] Huang, H., Wu, N., Liang, Y., Peng, X. & Shu, J. SLNL: A novel method for gene selection and phenotype classification. *Int. J. Intell. Syst.* **37**(9), 6283–6304. 10.1002/int.22844 (2022).

[37] Bilal, A. et al. Advanced CKD detection through optimized metaheuristic modeling in healthcare informatics. *Sci. Rep.* **14**(1), 12601 (2024). 38824162 10.1038/s41598-024-63292-5 PMC11144271

[38] Bilal, A., Alarfaj, F. K., Khan, R. A., Suleman, M. T. & Long, H. m5c-iEnsem: 5-methylcytosine sites identification through ensemble models. *Bioinformatics* **41**(1), btae722 (2024). 10.1093/bioinformatics/btae722 PMC11911556 39657957

[39] Talukder, M. S. H., Chowdhury, M. R., Sourav, M. S. U., Al Rakin, A., Shuvo, S. A., Sulaiman, R. B., ... & Haque, Z. (2023). JutePestDetect: An intelligent approach for jute pest identification using fine-tuned transfer learning. *Smart Agricultural Technology*, *5*, 100279.

[40] Khan, S., Sajjad, M., Hussain, T., Ullah, A. & Imran, A. S. A review on traditional machine learning and deep learning models for WBCs classification in blood smear images. *Ieee Access* **9**, 10657–10673 (2020).

[41] Saraswat, M. & Arya, K. V. Automated microscopic image analysis for leukocytes identification: A survey. *Micron* **65**, 20–33 (2014). 25041828 10.1016/j.micron.2014.04.001

[42] Deshpande, N. M., Gite, S. & Aluvalu, R. A review of microscopic analysis of blood cells for disease detection with AI perspective. *PeerJ Computer Science* **7**, e460 (2021). 33981834 10.7717/peerj-cs.460 PMC8080427

[43] Sajjad, M. et al. Leukocytes classification and segmentation in microscopic blood smear: a resource-aware healthcare service in smart cities. *IEEE Access* **5**, 3475–3489 (2016).

[44] Pešić, I., 2020. Segmentation and Classification of Leucocyte Images for Detection of Acute Lymphoblastic Leukemia. In *2020 7th ETRAN&IcETRAN international conference [Internet]. Belgrade: IcETRAN* (2–7).

[45] Luan, S. et al. Deep learning for fast super-resolution ultrasound microvessel imaging. *Phys. Med. Biol.* **68**(24), 245023. 10.1088/1361-6560/ad0a5a (2023). 10.1088/1361-6560/ad0a5a 37934040

[46] Tran, T., Kwon, O.H., Kwon, K.R., Lee, S.H. and Kang, K.W., 2018, December. Blood cell images segmentation using deep learning semantic segmentation. In *2018 IEEE international conference on electronics and communication engineering (ICECE)* (13–16). IEEE.

[47] Das, P. K., Meher, S., Panda, R. & Abraham, A. An efficient blood-cell segmentation for the detection of hematological disorders. *IEEE Transactions on Cybernetics* **52**(10), 10615–10626 (2021). 10.1109/TCYB.2021.3062152 33735090

[48] Savkare, S.S. and Narote, S.P., 2015, December. Blood cell segmentation from microscopic blood images. In *2015 International conference on information processing (ICIP)* (502–505). IEEE.

[49] Al-Hafiz, F., Al-Megren, S. & Kurdi, H. Red blood cell segmentation by thresholding and Canny detector. *Procedia Computer Science* **141**, 327–334 (2018).

[50] Nee, L. H., Mashor, M. Y. & Hassan, R. White blood cell segmentation for acute leukemia bone marrow images. *Journal of Medical Imaging and Health Informatics* **2**(3), 278–284 (2012).

[51] Jia, Y., Chen, G. & Chi, H. Retinal fundus image super-resolution based on generative adversarial network guided with vascular structure prior. *Sci. Rep.* **14**(1), 22786 (2024). 39354105 10.1038/s41598-024-74186-x PMC11445418

[52] Zhang, M., Li, X., Xu, M. & Li, Q. Automated semantic segmentation of red blood cells for sickle cell disease. *IEEE J. Biomed. Health Inform.* **24**(11), 3095–3102 (2020). 32749972 10.1109/JBHI.2020.3000484

[53] Zheng, X., Wang, Y., Wang, G. & Liu, J. Fast and robust segmentation of white blood cell images by self-supervised learning. *Micron* **107**, 55–71 (2018). 29425969 10.1016/j.micron.2018.01.010

[54] Xiang, F. et al. Multimodal Masked Autoencoder Based on Adaptive Masking for Vitiligo Stage Classification. *J. Imaging Inform. Med.*10.1007/s10278-025-01521-7 (2025). 40301294 10.1007/s10278-025-01521-7 PMC12920979

[55] Xu, X. et al. Large-field objective lens for multi-wavelength microscopy at mesoscale and submicron resolution. *Opto-Electronic Adv.* **7**(6), 10.2902/oea.2024.230212 (2024).

[56] Long, T., Song, X., Han, B., Suo, Y. & Jia, L. In situ magnetic field compensation method for optically pumped magnetometers under three-axis nonorthogonality. *IEEE Trans. Instrum. Meas.* **73**, 1–12. 10.11096/TIM.2023.3331425 (2024).

[57] Zhang, C. et al. Clot removAl with or without decompRessive craniectomy under ICP monitoring for supratentorial IntraCerebral Hemorrhage (CARICH): a randomized controlled trial. *Int. J. Surg.* **110**(8), 4804–4809. 10.1097/JS9.0000000000001466 (2024). 38640513 10.1097/JS9.0000000000001466 PMC11325930

[58] Jiang, C. et al. Xanthohumol Inhibits TGF-β1-Induced Cardiac Fibroblasts Activation via Mediating PTEN/Akt/mTOR Signaling Pathway. *Drug Design, Dev. Therapy* **14**, 5431–5439. 10.2147/DDDT.S282206 (2020). 10.2147/DDDT.S282206 PMC7732164 33324040

[59] He, W. et al. Neuromorphic-enabled video-activated cell sorting. *Nat. Commun.* **15**(1), 10792. 10.1038/s41467-024-55094-0 (2024). 39737963 10.1038/s41467-024-55094-0 PMC11685671

[60] Jiang, M., Cheng, L., Qin, F., Du, L. & Zhang, M. White blood cells classification with deep convolutional neural networks. *Int. J. Pattern Recognit Artif Intell.* **32**(09), 1857006 (2018).

[61] Praveen, N., Punn, N.S., Sonbhadra, S.K., Agarwal, S., Syafrullah, M. and Adiyarta, K., October. White blood cell subtype detection and classification. In: *2021 8th International Conference on Electrical Engineering, Computer Science and Informatics (EECSI)* (203–207). (IEEE, 2021)

[62] Gautam, A., Singh, P., Raman, B. and Bhadauria, H., 2016, November. Automatic classification of leukocytes using morphological features and naïve Bayes classifier. In *2016 IEEE region 10 conference (TENCON)* (1023–1027). IEEE.

[63] Macawile, M.J., Quiñones, V.V., Ballado, A., Cruz, J.D. and Caya, M.V. White blood cell classification and counting using convolutional neural network. In: *2018 3rd International conference on control and robotics engineering (ICCRE)* (259–263). (IEEE, 2018)

[64] Bani-Hani, D., Khan, N., Alsultan, F., Karanjkar, S. and Nagarur, N., 2018, October. Classification of leucocytes using convolutional neural network optimized through genetic algorithm. In: *Proc. of the 7th Annual World Conference of the Society for Industrial and Systems Engineering*.

[65] Kutlu, H., Avci, E. & Özyurt, F. White blood cells detection and classification based on regional convolutional neural networks. *Med. Hypotheses* **135**, 109472 (2020). 31760248 10.1016/j.mehy.2019.109472

[66] Sharma, S., Gupta, S., Gupta, D., Juneja, S., Gupta, P., Dhiman, G. and Kautish, S., 2022. Deep learning model for the automatic classification of white blood cells. Computational Intelligence and Neuroscience, 2022. 10.1155/2022/7384131 PMC8769872 35069725

[67] Su, M.C., Cheng, C.Y. and Wang, P.C., 2014. A neural-network-based approach to white blood cell classification. *Sci. world J*. 2014. 10.1155/2014/796371 PMC3929189 24672374

[68] Sudha, D. and Ramakrishna, M., 2017, March. Comparative study of features fusion techniques. In *2017 International Conference on Recent Advances in Electronics and Communication Technology (ICRAECT)* (235–239). IEEE.

[69] Dong, N., Feng, Q., Zhai, M., Chang, J. and Mai, X., 2023. A novel feature fusion based deep learning framework for white blood cell classification. *J. Ambient Intell. Humanized Comput. * 1–13.

[70] Loey, M., Naman, M. & Zayed, H. Deep transfer learning in diagnosing leukemia in blood cells. *Computers* **9**(2), 29 (2020).

[71] Tamang, T., Baral, S. & Paing, M. P. Classification of white blood cells: A comprehensive study using transfer learning based on convolutional neural networks. *Diagnostics* **12**(12), 2903 (2022). 36552910 10.3390/diagnostics12122903 PMC9777002

[72] Vogado, L. H., Veras, R. M., Araujo, F. H., Silva, R. R. & Aires, K. R. Leukemia diagnosis in blood slides using transfer learning in CNNs and SVM for classification. *Eng. Appl. Artif. Intell.* **72**, 415–422 (2018).

[73] Saleem, S., Amin, J., Sharif, M., Anjum, M.A., Iqbal, M. and Wang, S.H., 2021. A deep network designed for segmentation and classification of leukemia using fusion of the transfer learning models. Complex & Intelligent Systems, pp.1–16.

[74] Yang, S.K., You, F.C. and Sun, D.Z., 2023, April. Multi-classification recognition of blood cell images based on transfer learning. In *Third International Conference on Artificial Intelligence and Computer Engineering (ICAICE 2022)* (12610, 300–305). SPIE.

[75] Saidani, O. et al. White blood cells classification using multi-fold pre-processing and optimized CNN model. *Sci. Rep.* **14**(1), 3570 (2024). 38347011 10.1038/s41598-024-52880-0 PMC10861568

[76] Dwivedi, K. & Dutta, M. K. Microcell-Net: A deep neural network for multi-class classification of microscopic blood cell images. *Expert. Syst.* **40**(7), e13295 (2023).

[77] Fırat, H. Classification of microscopic peripheral blood cell images using multibranch lightweight CNN-based model. *Neural Comput. Appl.* **36**(4), 1599–1620 (2024).

[78] Siddique, M. A. I., Aziz, A. Z. B. & Matin, A. An improved deep learning based classification of human white blood cell images. In 2020 11th International Conference on Electrical and Computer Engineering (ICECE), 149–152 (IEEE, 2020)

[79] Rustam, F. et al. White blood cell classification using texture and RGB features of oversampled microscopic images. *Healthcare* **10**, 2230 (2022). 36360571 10.3390/healthcare10112230 PMC9691098

[80] Özyurt, F. A fused CNN model for WBC detection with MRMR feature selection and extreme learning machine. *Soft. Comput.* **24**, 8163–8172 (2020).

[81] Girdhar, A., Kapur, H. & Kumar, V. Classification of white blood cell using convolution neural network. *Biomed. Signal Process. Control* **71**, 103156 (2022).

[82] Acevedo, Andrea; Merino, Anna; Alférez, Santiago; Molina, Ángel; Boldú, Laura; Rodellar, José (2020), “A dataset for microscopic peripheral blood cell images for development of automatic recognition systems”, Mendeley Data, V1, 10.17632/snkd93bnjr.1 10.1016/j.dib.2020.105474 PMC7182702 32346559

[83] Reza, A. M. Realization of the contrast limited adaptive histogram equalization (CLAHE) for real-time image enhancement. *Journal of VLSI signal processing systems for signal, image and video technology* **38**, 35–44 (2004).

[84] Taspinar, Y. S. Light weight convolutional neural network and low-dimensional images transformation approach for classification of thermal images. *Case Studies in Thermal Engineering* **41**, 102670 (2023).

[85] Mei, L. et al. High-accuracy and high-throughput reactive lymphocyte identification using lightweight neural networks. *Biomed. Signal Process. Control* **97**, 106722 (2024).

[86] Abbas, A., Abdulrazaq, M.B. and Adel, A.Z., 2025. Efficient Lightweight CNN for Automated Classification of B-cell Acute Lymphoblastic Leukemia. *Comput. Biol. Chem.*, 108645. 10.1016/j.compbiolchem.2025.108645 40845691

